# Microfluidic Applications of Artificial Cilia: Recent Progress, Demonstration, and Future Perspectives

**DOI:** 10.3390/mi13050735

**Published:** 2022-05-03

**Authors:** Vignesh Sahadevan, Bivas Panigrahi, Chia-Yuan Chen

**Affiliations:** 1 Department of Mechanical Engineering, National Cheng Kung University, Tainan 701, Taiwan; n18097013@mail.ncku.edu.tw; 2 Department of Refrigeration, Air Conditioning and Energy Engineering, National Chin-Yi University of Technology, Taichung 411, Taiwan; bivas@ncut.edu.tw

**Keywords:** artificial cilia, microfluidics, flow manipulation, biological/medical applications

## Abstract

Artificial cilia-based microfluidics is a promising alternative in lab-on-a-chip applications which provides an efficient way to manipulate fluid flow in a microfluidic environment with high precision. Additionally, it can induce favorable local flows toward practical biomedical applications. The endowment of artificial cilia with their anatomy and capabilities such as mixing, pumping, transporting, and sensing lead to advance next-generation applications including precision medicine, digital nanofluidics, and lab-on-chip systems. This review summarizes the importance and significance of the artificial cilia, delineates the recent progress in artificial cilia-based microfluidics toward microfluidic application, and provides future perspectives. The presented knowledge and insights are envisaged to pave the way for innovative advances for the research communities in miniaturization.

## 1. Introduction

Biological cilia are hair-like microscopic structures found on the outer surfaces of nearly every mammalian cell. These microscopic structures allow the cells to interact and sense their surrounding environment [1]. The length of cilia usually varies between 2–15 µm, and they possess complex internal structures comprised of outer doublet microtubules, central microtubules (Axoneme), and dynein arms, which further determines cilia’s function as well as their motion. According to their functions, biological cilia can be broadly classified into two major groups: motile and non-motile, or primary cilia [2]. The cilia could be identified, whether motile or primary, using the arrangements of nine pairs of two microtubules with two central pair-structure known as axonemes. In general, if the cilium is with nine pairs of two microtubules but without two-central pair apparatus, the cilium is widely considered as primary cilium. On the other hand, if the cilium has nine pairs of two peripheral microtubules with two central pair microtubules, the arrangement is considered a motile cilium [3,4]. Motile cilia exhibit spatial, temporal, and even oriental asymmetry in their motion to generate flow around them. In contrast, the non-motile cilia are stationary by nature and act as a sensor. Applications of motile cilia are immense. Motile cilia on the outer membrane paramecia help them propel in the fluid 10 times faster than their body length. Additionally, the cilia on the outer surface of the juvenile starfish allow them to select food by creating vortices around them [5,6]. Non-motile cilia can be found on the kidney tubule, where they sense the direction of urine flow and direct the cells accordingly. The motile cilia exhibit a planar motion and beat in a straight path during the forward stroke and roll back to their original position by moving close to the surface in a tangential manner during the recovery stroke. This spatial asymmetry can generate a substantial flow around the cells [1]. Similarly, cilia on the embryo nodal cell exhibit a tilted conical beating path that produces the fluid flow to determine further left-right symmetry in the body [7,8].

Microfluidics is the science of manipulating the amount of fluid with channels, where at least one dimension is generally in a range of 10~1000 µm [9]. Microfluidics exploits its most apparent characteristic, such as its miniaturized size to efficiently handle a small amount of fluid in a minimal time scale. The flow physics of microfluidics is purely laminar, and the viscous force dominates over the inertial force in the flow regime. Hence, it is quite challenging to manipulate the fluid flow in the microfluidic environment due to the effect of the viscous force. As discussed earlier, nature provides an ingenious way for the microorganisms and eukaryotic cells to manipulate the flow around cells by means of cilia. Taking inspiration from the natural cilia, artificial cilia have now been fabricated in the laboratory environment to manipulate the fluid flow within the microfluidic environment. The applications and usages of these artificial cilia-based microfluidic devices are unparalleled.

In the past two decades, the development of microfluidics and artificial cilia research has increased drastically. The existing reviews on microfluidic based devices focused on any specific topics. For instance, the studies were focused on particular actuation techniques like magnetics [10,11,12,13,14] and light [15], specific applications like mixing [12], robotics [16] and particle manipulation [11,17,18], or fabrication techniques like 3D printing [19]. Few excellent reviews are presented in the field of artificial cilia-based microfluidic devices [20,21]. These reviews have extensively discussed the principles of artificial cilia, fabrication processes, actuation mechanisms, modeling of their motion, etc. Along with these fundamental aspects, this review article has put particular emphasis on describing various biological as well as the industrial applications of these artificial cilia-based microfluidic devices. This will not only bridge the existing knowledge gap in the field of artificial-based microfluidics but will also provide a perspective towards future applications and possible research directions. The arrangement of the article is delineated as follows. First, the importance of the natural cilia dynamics was discussed, followed by the description of the fabrication techniques for artificial cilia and the respective actuation methodologies. In the following sections, dynamic beating behaviors of artificial cilia and their applications in microfluidics were also illustrated. Their roles in contemporary applications were discussed. Finally, in view of the general summary, an outlook on conclusions and future perspectives was offered.

## 2. From Natural Cilia to Artificial Cilia

Natural cilia are hair-like structures that perform the operations such as feeding, swimming, transporting, moving, and sensing functions in almost all cell types [22,23,24,25]. In motile natural cilia category, their applications in coral reef [22] and the respiratory tract [23] towards particle transportation is exemplary. The natural cilia in the coral surfaces generate metachronal wave motion to create flows using the asymmetric dynamic beating behavior. Due to this flow behavior, the materials such as oxygen and nutrients are transported without the typical wave streams [22]. The natural cilia shielded on the lumen within the human respiratory tract transport the dust and bacteria to remove towards the oropharynx [23]. Natural cilia in the Cactus spines and trachea are well regarded for their directional transporting ability due to their asymmetric motion and anisotropic surface. Beating natural cilia with amorphous sheets and dense tufts can create the propulsion forces which facilitate the locomotion in many species [26,27,28]. Depending upon the size of the organisms, the functions of the natural cilia vary. For example, including the surface of starfish larvae, many organismic ciliary structures were evolved towards facilitating their feeding. The non-motile natural cilia perform sensing operations and adapt to the ambient atmosphere [23]. They were used for sensing work to defend the predators and sense the food and atmosphere in the Animalia kingdom. The natural cilia in the spider tarsi legs sense air flow and other vibrations. The external vibration makes the natural cilia bend where the electrical impulses create and reach the spiders [23].

Motivated by these biological cilia, congeners such as artificial cilia have been advanced and exploited in microfluidics and micro/nanorobotics to realize performances of propelling, transporting, moving, and mixing [29,30,31,32,33,34,35]. Even though the in-depth information and uses of artificial cilia in transportation, moving, and sensing will be seen in the upcoming chapters, the insights into the evolvement of artificial cilia from natural cilia are explained here by detailing their participation with examples of each significant application. Wang et al. [15] fabricated magnetically actuated cilia using Polydimethylsiloxane (PDMS) and cobalt powder by biomimicking cactus spines and trachea cilia in favor of transportation. The artificial cilia were capable of transporting hydrogel slices directionally using their anisotropic surface and asymmetric motion. The asymmetric stroke of the artificial cilia in the sequential magnetic field drove the hydrogel forward. The asymmetric beating behavior and metachronal wave motion are two intriguing benefits exploited using artificial cilia [16,28,29]. The nonreciprocal or asymmetric beating behavior was achieved by creating the difference in the swept area of forward and recovery strokes of artificial cilia [30]. The nonreciprocal motion was measured by the difference between the swept area of the artificial cilia tip and the swept area of the semicircle. The semicircle was created by the artificial cilia length as the radius. The direction of the particle transportation can be adjusted by changing the levels of forward and recovery strokes [31]. The out-of-phase behavior or the phase difference in the neighboring artificial cilia raised the oscillating waves above the surface of the artificial cilia, known as metachronal wave motion. Another study was reported [34] in favor of transportation, in which the photo-actuated artificial cilia were obtained by mimicking the Paramecium aurelia’s complex mechanical functions and surface responsiveness. The light-controllable cilia were fabricated using Diarylethene and are capable of transporting objects, demonstrated to transport the polystyrene bead (PB) of 1 mm in diameter. Recent bioinspired artificial cilia established from natural cilia for transporting ability are reported elsewhere [23,30,31,34,36,37,38,39,40]. Inspired by the starfish surface, researchers designed ciliary bands [29], which can be actuated by ultrasound. The ciliary band was successfully demonstrated for locomotion, trapping polystyrene particles, and transportation of water droplets.

The biomimetic inventions have not ended here—the establishments are also reflected in sensing artificial cilia. For sensing, conceptual-wise, the working principle of the artificial was designed similar to the working principle of natural cilia. Artificial cilia for sensing have two sections. The first section is the cilia part where typical magnetic particles (Carbonyl iron powder (CIP) and, neodymium-iron-boron (NdFeB)), PDMS, glass fibers, and polymer materials were used. The second section is the signal processing section. Graphene nanoplatelets [41], carbon nanotubes (CNTs), SiO_2_, carbon nanofiber, iron nanowires, and AgNW were employed as the sensing/signal processing part [41,42,43,44,45,46,47,48]. Signals such as the piezoelectric and piezoresistive types were created due to cilia bending and processed through the signal processing units [41]. For instance, in the study [42], the high aspect ratio (HAR) artificial cilia were fabricated by replicating goldfish’s neuromasts and arthropod filiform hair. The HAR cilia sensor was comprised of PDMS cilia structure, and graphene nanoplatelet infused microchannel. The change in resistance of graphene nanoplatelet was realized in the presence of external flow or touch over the PDMS cilia sensor.

## 3. Fabrication Techniques for Artificial Cilia

### 3.1. Micro-Molding Fabrication Techniques

**Figure 1:**
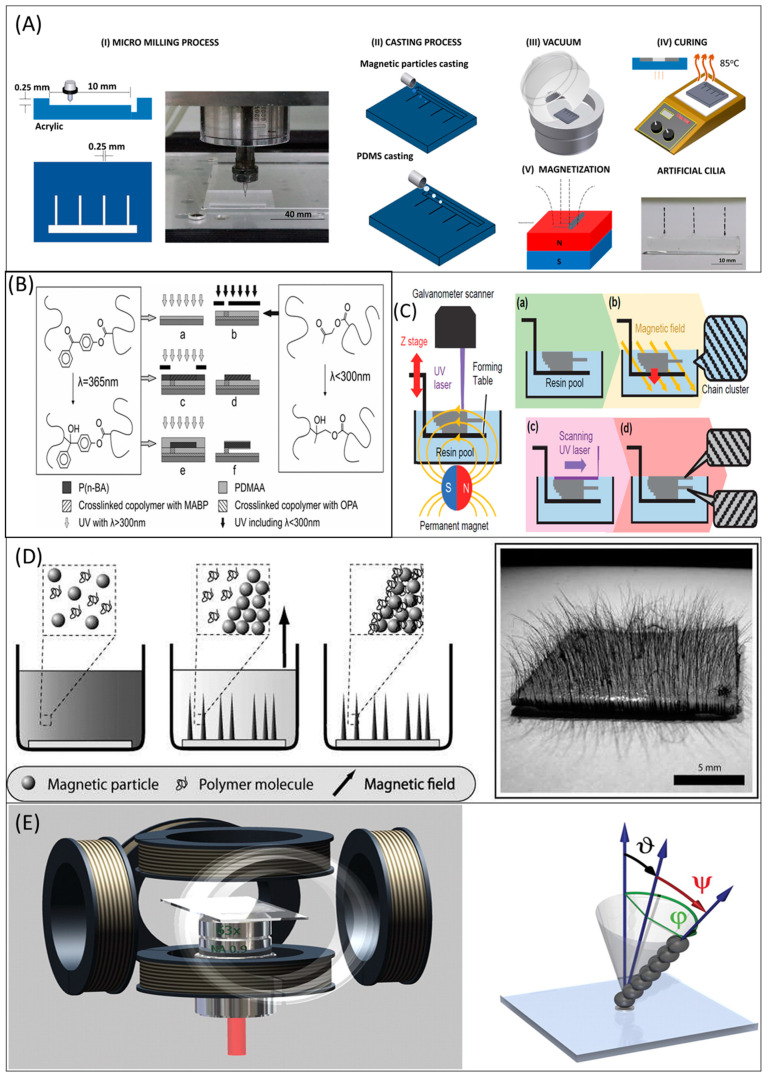
Fabrication techniques for artificial cilia. (**A**) The micro-molding fabrication process demonstrated fabricating multi-segmented magnetic artificial cilia using the following fabrication processes: (**i**) cilia shape patterning in acrylic sheet, (**ii**) magnetic and PDMS mixture pouring in the pattern, (**iii**) cured in the hot surface plate at 85 °C for 48 h, and (**iv**) procedures of peeling-off artificial cilia from the acrylic substrate and magnetization. The figure was reproduced with permission from [55], under a Creative Commons BY Non-Commercial No Derivative Works (CC BY-NC-ND 4.0) license, published by Elsevier, 2021. (**B**) Photolithography fabrication process depicted fabricating magnetic artificial cilia using the fabrication following processes: (**i**) substrate preparing, (**ii**) anchor preparing, (**iii**) cilia body layer preparing, (**iv**) ciliary shape developing, (**v**) cilia coating and (**vi**) peeling-off. The figure was reproduced with permission from [74], published by John Wiley and Sons, 2011. (**C**) 3D printing fabrication process illustrated to manufacture magnetic artificial cilia. The 3D printing technique was based on stereolithography. Initially, the resin material was poured, and the UV laser of 355 nm was employed to cure. The cilia body were magnetized using a permanent magnet. The surface of the substrate was set at the level of the layer. The substrate table went down to form the next layer after the curing process of the previous layer, and the process was repeated until the desired shape was achieved. The figure was reproduced with permission from [68], published by the Society of Photopolymer Science and Technology (SPST), 2018. (**D**) The facile bottom-up approach in which elastomeric poly (styrene-block-isoprene-block-styrene), toluene, and magnetic particles were mixed, and the mixture was ultrasonicated. A PTFE dish was placed in the aqueous medium (**left**). The setup was kept in the magnetic field. As a result of the magnetic field, the conical structure was formed by magnetic particles, but the polymer was still in the suspensions (**middle**). Toluene evaporated the suspension when the polymer material covered the empty holes between the magnetic particles and artificial cilia were fabricated (**right**). The figure was reproduced with permission from [69], published by the American Chemical Society, 2010. (**E**) Self-assembly approach in which three magnetic coils were arranged orthogonally to render the magnetic field. The artificial cilium was created by assembling the superparamagnetic particles due to the magnetic field (**right**). The figure was reproduced with permission from [59], published by the National Academy of Sciences, 2010.

The magnetically actuated artificial cilia fabricated by means of micro-molding techniques required a less complex fabrication process and considered precise by nature. This fabrication technique is one of the template-based fabrication techniques. The micro-mold fabrication process involves four critical steps. The first step is to pattern the mold corresponding to the artificial cilia. The second process introduces the polymer material to the pattern. The third step is arranged for the solidification of the pattern. The final step is separating or peeling off the artificial cilia. Chen and his team [49,50,51,52,53,54,55] fabricated a wide range of artificial cilia with universality using micro-molding fabrication processes. For instance, Wu et al. [56] demonstrated a micro-molding fabrication process that involves a series of computerized numerical control (CNC) micromachining processes towards preparing the mould for artificial cilia., PDMS-magnetic composite casting was carried out followed by PDMS casting to create the structure of artificial cilia and its microfluidic environment. Following the PDMS casting, the sample was kept in the hot plate for curing at 90 °C before secluding the artificial cilia [56]. The critical challenge coming up with magnetic artificial cilia is their size, as they are considered relatively oversized than the natural cilia. Recently, the magnetic artificial cilia [57] were showcased of the same size (Radius = 200 nm, Length = 6µm) as their counterpart (i.e., biological cilia). The proposed magnetic artificial cilia were fabricated using a tailored molding process. Figure 1A illustrates the use of micro-molding fabrication process towards the fabrication of multi-segmented magnetic artificial cilia. The fabrication was carried out following processes such as: (i) artificial cilia were patterned in an acrylic sheet, (ii) magnetic and PDMS mixture were poured into the pattern, (iii) the pattern was cured on the hot plate at 85 °C for 48 h, (iv) procedures of peeling-off artificial cilia from the acrylic substrate and the following magnetization of the artificial cilia.

### 3.2. Photolithography Fabrication Techniques

The photolithography fabrication technology was used to fabricate soft patterned thin film on the substrate using light. In general, UV light is a popular alternative to this technique. Still, various lights with different wavelengths, such as X-rays, visible light, and extreme UV rays, were also employed depending on the requirements. In a study [58] where a two-step lithography process was employed to fabricate nickel-iron (Ni-Fe) permalloy-based artificial cilia by the researchers. In the first step, Cu’s sacrificial layer was sputtered on the negative photoresist material (NR9 1500Py Futurex), followed by the Ni-Fe layer. The desired thickness of the artificial cilia was defined by the deposition of Ni-Fe. The ciliary structures were formed after removing the photoresist material using acetone. In the second step of lithography, the ciliary structures were pinned on the glass substrate using the Ti anchor to hold the cilia on the substrate. Then, the sacrificial Cu layer was removed by 5% ammonium hydroxide solution.

The photolithography fabrication technique is preferred over some other fabrication techniques for the following reasons. The first reason is that this technique offers high precision. In addition, this method is highly controlled together with high throughput, compared to the bead self-assembly [59,60], which required additional control [58]. The recent discussions and approaches over photolithography processes to fabricate artificial cilia can be found elsewhere [29,44,61,62]. Figure 1B illustrates the photolithography fabrication process depicted fabricating magnetic artificial cilia following processes such as: (i) substrate preparing, (ii) anchor preparing, (iii) cilia body layer preparing, (iv) ciliary shape developing, (v) cilia coating, and (vi) peeling-off.

### 3.3. 3D/4D/5D Printing Fabrication Techniques

Previously explained, micro-molding and photolithography fabrication technologies are limited because different molds need to be used for different designs in the micro-molding fabrication process. The photolithography fabrication technology requires photoresist, external light sources, the repeatability of the lithography process, and the cleanroom fabrication setup. In the 3D printing technique eliminates these extensive experiment setups and various sizes of artificial cilia could be made without fabrication complexity. A 3D CAD diagram was used to design the three-dimensional artificial cilium to fabricate it under computer programming [63]. The 3D printing technology for artificial cilia was well illustrated by Liu et al. [64]. Recent discussions and approaches over 3D printing technology (Figure 1C) to fabricate artificial cilia can be found in some recent articles [41,42,46,65].

In 2004, 4D printing technology for multi-material [66] was proposed by by S. Tibbits. In the 4D printing technique, the programmed multi-materials can morph their shape over a period of time, even after they came out of the printer. Recently, 4D printing of artificial cilia was proposed by Tsumori et al. [67]. The printing system printed out the 3D artificial cilia whose anisotropy could be changed simultaneously. Next to 4D printing, 5D-printing was proposed by Tsumori et al. [68] for the magnetic artificial cilia. In this process, three design parameters (x, y, z) were used to fabricate the artificial cilia’s shape. Two more parameters (θ, ψ) were utilized in aligning magnetic chain clusters. Five design parameters (x, y, z, θ, ψ) were optimized simultaneously.

### 3.4. Facile Bottom-Up Approaches

The facile bottom-up approach is one of the template-free fabrication methods which is unconstrained and the artificial cilia made by this process is highly flexible by nature. This approach involves simple fabrication and operating steps. Timonen et al. [69] demonstrated the facile bottom-up approach (Figure 1D) to fabricate the magnetic artificial cilia. The magnetic particle such as cobalt was mixed with the solvent toluene and elastomeric poly (styrene-block-isoprene-block-styrene) polymer. Poly (tetrafluoroethylene) (PTFE) was used as the substrate in the study. The suspension was ultrasonicated for 10 s to have the high aspect magnetic artificial cilia. In addition, the bottom-up approach was used to fabricate the minimal model system [70] comprised of microtubule (MT) bundles and molecular motors. The exemplified minimal model system was demonstrated to resemble the beating behaviors of the eukaryotic cilia and flagella by self-assembling the MTs and molecular motors.

### 3.5. Roll-Pulling Approaches

The roll-pulling approach was showcased by the researchers in the study [71] where the custom-made setup was comprised of aluminum roll, substrate, and the precursor medium. The aluminum roll was surrounded by magnetic pillars fabricated by soft lithography. The aluminum roll and glass substrate were separated in a particular gap and rotated with a constant line speed with the help of a rigid string to eliminate friction. The glass substrate was carried the precursor medium comprised of magnetic particles and PDMS. The precursor medium was then pulled by the pre-molded magnetic pillars during the roll rotation and became filaments of a certain length before breaking. The dimensions of magnetic filaments can be adjusted by the size of micro-pillars and the line speed of the roll and substrate. The proposed fabrication technique has advantages over other fabrication techniques in terms of scaling down the dimension of artificial cilia. For example, the 3D printing technology [42] for PDMS artificial cilia is challenging to fabricate high aspect ratio cilia. It required additional attention to print out PDMS polymer materials for microchannels and biomimetic structures (with less than 150 μm diameter) due to its size limitation.

### 3.6. Self-Assembly Fabrication Techniques

Wang et al. [60] demonstrated a new affordable in-situ fabrication process named the self-assembly technique. In this fabrication process, micro-sized beads were self-assembled and constructed to form the artificial cilia and encapsulated with soft polymer coatings. The self-assembly approach for the artificial cilia has been illustrated in Figure 1E [59]. The self-assembly approach was comprised of three electromagnets. The three electromagnets were arranged orthogonally to direct the applied magnetic field. The superparamagnetic particles were assembled to create the artificial cilium due to the controlled magnetic field.

### 3.7. Field-Effect Spinning Approaches

High sensing performance can be exploited by developing artificial cilia with identical sensory functions. The field-effect spinning (FES) method was reported in the study [72] for the fabrication of artificial cilia. In the previously reported conventional approach, the fiber filaments were pulled without direction. In contrast to the above-mentioned approach, the FES method was demonstrated to produce the vertical and uniformly sized artificial cilia.

### 3.8. Dip-Coating Fabrication Techniques

The dip-coating fabrication technique can be used for the fabrication of large size ciliary structures. High aspect ratio shapes can be fabricated using this approach with the following of four steps. In the first step, to give the shape to the ciliary structure, the metals like stainless steel pins were used along with the dielectric material. In the second step, the fixture, the dielectric material, and the spaces between the cilia were covered by rubber shims. In the third step, the structure was removed from the downside, and the dielectric material followed the structure of the previously placed fixture immediately. In the fourth step, the downside channels were sealed. The step-by-step fabrication processes along flow charts can be found in the study [73].

## 4. Artificial Cilia Actuation Methodologies

### 4.1. Optical Actuation Techniques

**Figure 2:**
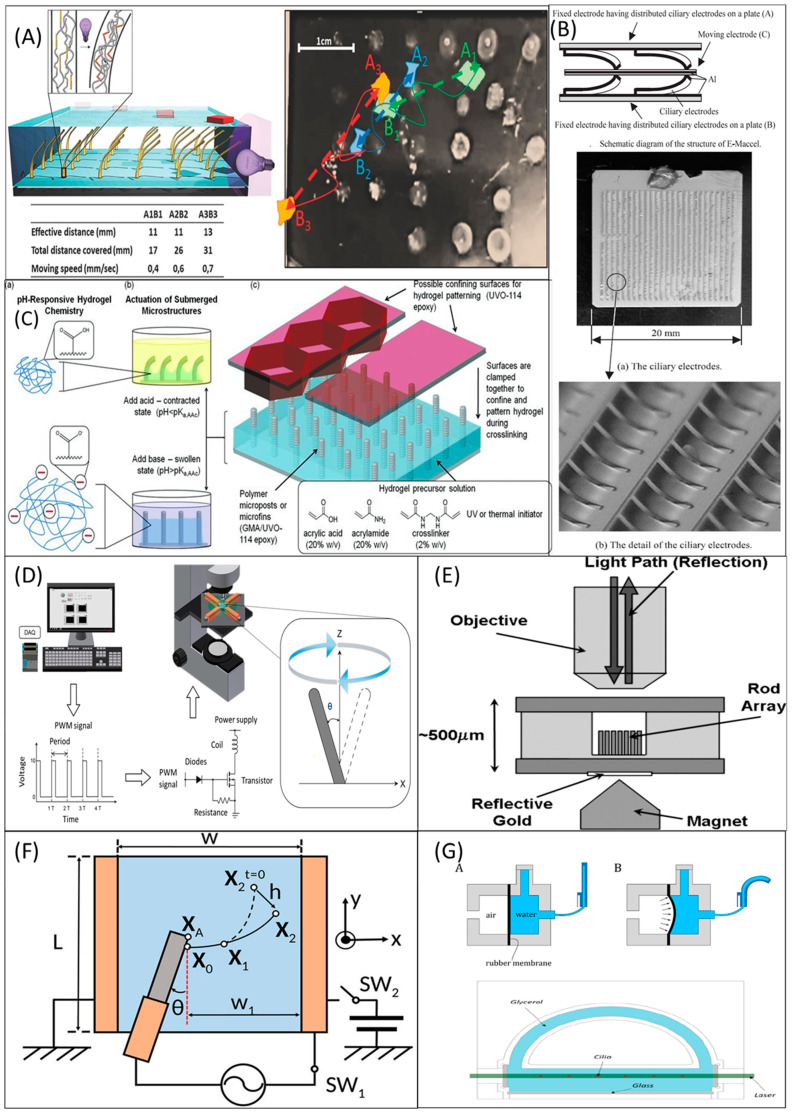
Artificial cilia actuation methodologies. (**A**) Optic actuation in which the photoresponsive polymers transported objects by having bending motion as responsive towards the light (**left**). The curved lines were the trajectory paths on which the objects were transported, and the dashed lines were the effective linear distance the objects covered (**right**). The figure was reproduced with permission from [77], published by John Wiley and Sons, 2016. (**B**) The electrostatic actuation setup comprised two fixed electrodes, one moving electrode, and multiple ciliary electrodes (**top**). The electrostatic force was released in the spaces between moving and ciliary electrodes when the applied voltage was exerted in between the fixed and moving electrode. Due to the electrostatic force, the moving electrode moved towards the fixed electrode parallel and perpendicularly. The figure was reproduced with permission from [80], published by the Institute of Electrical Engineers, 2004. (**C**) The schematics of pH actuation process for hydrogel artificial cilia. The hydrogel response towards pH was shown (**left**). The figure was reproduced with permission from [83], published by John Wiley and Sons, 2011. (**D**) The schematics of electromagnetic actuation for artificial cilia. The algorithm was fed into the system to create pulse width modulation through which the artificial cilia were actuated using electromagnet coils. Inset: The beating trajectory of the artificial cilia. The figure was reproduced with permission from [56] under a Creative Commons BY (CC BY) license, published by MDPI, 2017. (**E**) Permanent magnetic actuation in which the permanent magnet of 0.5 T magnetic field was used to actuate the nanorod artificial cilia. The magnetic field was sufficient to generate the deflection up to 7 µm, equivalent to 20° of bending angle of artificial cilia. The figure was reproduced with permission from [100], published by the American Chemical Society, 2007. (**F**) ICEO artificial cilia actuation using the electric field (details of the experimental setup were discussed previously in the Induced charge electro-osmosis using AC electric field section). The figure was reproduced with permission from [103] under a Creative Commons BY (CC BY) license, published by AIP, 2020. (**G**) The strategy of pneumatic actuation (details of the experimental setup were discussed previously in the pneumatic actuation section). The figure was reproduced with permission from [108], under a Creative Commons Attribution NonCommercial License 4.0 (CC BY-NC) license, published by the American Association for the Advancement of Science (AAAS), 2020.

Optical actuation is the wireless actuation technology preferred in autonomous wireless microsystems [75]. Optically actuated cilia were established from materials consisting of acrylates or methacrylates and liquid-crystal polymer entailing azobenzene dyes using inkjet printing technology [76]. The visible light had a wavelength of 455–550 nm, and ultraviolet light was used to get the desired bending and complex movements of the artificial cilia in the water. The optical actuation method for the artificial cilia has been illustrated in Figure 2A. The figure illustrates the light actuation process demonstrated by Broer et al. [77]. The artificial cilia were made by the cross-linked polymers functionalized with azobenzene. The polymer was responsive to light and prototyped to transport the particles in the aqueous medium.

### 4.2. Electrostatic Actuation Techniques

Electrostatic actuation is done by the force between the conducting electrically charged objects. The electrostatic force used to actuate the artificial cilia can be attractive or repulsive. Den Toonder et al. [78] demonstrated such artificial cilia, which had a length and width of 100 mm, and 20 mm, respectively, and was made of polyimide (PI) and chromium (Cr). The artificial cilia had been electrostatically actuated for microfluidic applications. The rapid ciliary motion of the produced cilia was suitable for delivering the fluid flow over 0.6 mm/s [78]. The recent straightforward method to find the fluid flow velocity can be found elsewhere [79]. The Electrostatic actuation method for the artificial cilia has been illustrated in Figure 2B [80]. The setup comprised one moving electrode in the center, sandwiched by multiple ciliary electrodes deposited on two fixed electrodes on the outside. The electrostatic force was released in the spaces between moving and ciliary electrodes when the applied voltage was exerted between the fixed and moving electrode. Due to the electrostatic force, the moving electrode displaced towards the fixed electrode in parallel as well as perpendicular manner.

### 4.3. pH Actuation Techniques

The pH actuation is predominantly used for hydrogel actuators. But the actuation of hydrogel cilia was not limited to pH alone [81]. Hydrogels can be swelled and shrank over 10% of their original volume due to specific stimuli such as temperature (T), light, pH, etc., [82]. It gets attention due to the recent development of microfluidics and MEMS, increasing the need for small devices to work in microscale platforms. Hydrogels are the 3D polymer networks with 99 wt % of water. Changing pH intensity was used to stimulus artificial cilia for hydrogel actuation [21,81]. In the study [83], hydrogel-actuated artificial cilia were fabricated using micropost arrays and microfins. The actuators can be bent and upright straight by treating acids and bases. The soft lithography technique is ideal for manufacturing hydrogel artificial cilia [84]. The schematics of the pH actuation process for hydrogel cilia is seen in Figure 2C.

### 4.4. Resonance Actuation Techniques

In the resonance actuation technique, the artificial cilia were actuated under the resonance of the lead– zirconate–titanate (PZT) microstage. The piezoelectric transducer was excited by a signal generator. Photolithography microfabrication technique and deep reactive ion etching (DRIE) were used to fabricate the master mold. PDMS polymer was filled in the mold to manufacture the artificial cilia [85,86]. The resonance-actuated cilia-assisted micromixers can provide an efficient uniform mixing performance than diffusion-and-vibration mixtures [86].

### 4.5. Magnetic Actuation

Many actuation techniques exist to actuate the artificial cilia to harness the desired motions and deliver specific applications. But most actuation techniques have their drawbacks hindering their applications in biological domain. This is the primary reason why the researchers have turned their attention to the magnetic actuation system. For example, the electrostatic actuation system cannot be used for biological fluid because it leads to electrolysis [21,87]. Similarly, water is necessary for the hydrogel actuation of artificial cilia. In the air, water molecules gets absorbed, which leads to a long response time of a few hours even for miniaturized cilia [82,88]. Artificial cilia can be actuated using a permanent magnet [68,89,90,91] or electromagnet [49,50,92,93,94,95]. Both symmetric and asymmetric motions can be obtained from artificial cilia under the influence of the magnetic stimuli [49,58,96,97,98,99].

#### 4.5.1. Electromagnetic Actuation

Chen et al. [98] demonstrated the electromagnetic actuation technique for the artificial cilia using the custom-built electromagnetic system with four magnetic coils to achieve real time actuation for various microfluidic applications. The Electromagnetic actuation method for the artificial cilia has been illustrated in Figure 2D [56]. The figure shows the electromagnetic actuation comprised of four solenoidal coils. The complete algorithm to actuate a series of artificial cilia using a single electromagnet can be found elsewhere [95].

#### 4.5.2. Permanent Magnetic Actuation

Rotating a permanent magnet is the alternative method requiring less effort and complexity towards artificial cilia actuation [100]. Hanasoge et al. [58] achieved the asymmetric beating of artificial cilia by rotating the permanent magnet. The study reported that magnetic actuation induced the forward stroke; recovery stroke by releasing the accumulated elastic force. The asymmetric beating was achieved by the interplay of elastic, magnetic, and viscous forces. Figure 2E illustrates permanent magnetic actuation in which the permanent magnet of 0.5 T magnetic field was used to actuate the nano-artificial cilia. The magnetic field was sufficient to generate the deflection up to 7 µm, equivalent to 20° of bending angle of artificial cilia.

### 4.6. Acoustic Actuation Techniques

The need for simple fabrication and straightforward actuation for the artificial cilia in lab-on-chip applications is in high demand. Orbay et al. [101] fabricated the artificial cilia using initial photolithography and UV polymerization technique which eliminated the complex magnetization process. The artificial cilia were actuated using the piezoelectric transducer (PZT) (81-7BB-27-4L0, Murata Electronics, Japan). The thin epoxy layer was used to connect the PZT to the optical path of the PDMS microchannel. The piezoelectric transducer was driven by the sine waves induced through the function generator. The RF amplifier (25A250A, Amplifier Research, USA) was used to amplify the sine waves. The artificial cilia were tested for the mixing operation where the fluorescein and DI water were used. It was found that the increment of mixing performance and complete mixing can be achieved by the voltage up.

### 4.7. Electric Stimulation Actuation Techniques

The electric stimulation actuation technique is practically advantageous for the remote and precise control of the artificial cilia. In this approach, the ciliary structures were made of dielectric material. The dielectric materials were polarized due to the dielectrophoresis in the alternating current (AC) electric field when the dielectric nanoparticles were aligned along the electric field direction, leading to the deformation of the artificial cilia. In the study [102], BaTiO_3_ was used as the dielectric nanoparticles along the PDMS cilia body. Dielectric fiber materials were used in a recent study [73]. By converting external physical cues into electric impulses using the piezoelectric or triboelectric effect principles, this actuation technique can also opt for sensors.

### 4.8. Induced Charge Electro-Osmosis Using AC Electric Field Techniques

The Induced charge electro-osmosis (ICEO) cilium was fabricated using a basic self-organizing process [103,104,105] demonstrated by Sugioka and the team. A graphite rod was immersed in the deionized water and intercalated between two Cu electrodes. As shown in Figure 2F, initially, *SW_1_* was turned off. *SW*_2_ was turned on by applying DC electric voltage (*V_o_*) between two Cu electrodes for duration *t_DC_* (30–120 s), which led to the formation of Carbon artificial cilia at position *X*_o_. The fabricated artificial cilia had a fibrous network which was identified using energy-dispersive x-ray spectroscopy (EDS). SW_1_ was turned on to actuate the artificial cilia by applying AC electric peak voltage between the graphite rod and the right Cu electrode at time *t_AC_,* when the non-fixed end of artificial cilia (*X*_o_) moved with the deflection range *h*. The prototyped artificial cilia were capable of producing asymmetric motions. Using the same ICEO and implementing AC electric field principle, a metachronal wave motion was showcased by actuating three artificial cilia with different lengths [106]. Recently, ICEO actuated artificial cilia were demonstrated to transport the square-shaped polyethylene object [107].

### 4.9. Pneumatical Actuation Techniques

**Figure 3:**
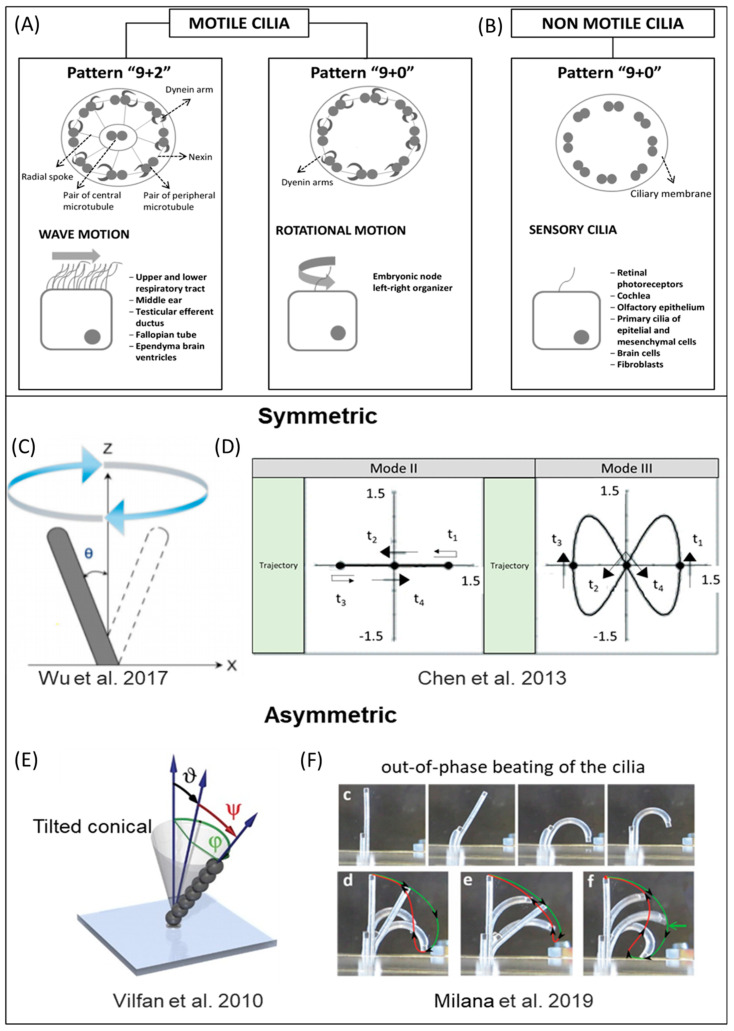
The dynamic beating behaviors of artificial cilia. (**A**) Structure and motion of motile biological cilia. The figure was adapted from [124] under a Creative Commons BY (CC-BY 4.0) license, published by IBIMA Publishing, 2015. (**B**) Structure and motion of primary biological cilia. The figure was adapted from [124] under a Creative Commons BY (CC-BY 4.0) license, published by IBIMA Publishing, 2015. The dynamic beating trajectory behaviors for artificial cilia were depicted as symmetric beating behavior [50,56] and asymmetric beating behavior [59,109] in schematics. (**C**) The symmetric dynamic beating trajectory of the artificial cilium has been illustrated by the 2D schematic. The figure was adapted from [56] under a Creative Commons BY (CC BY) license, published by MDPI, 2017. (**D**) Two different symmetric trajectories have been tested to analyze the mixing operation. The figure was reproduced with permission from [50], published by the Royal Society of Chemistry, 2013. (**E**) The superparamagnetic colloidal particles were assembled to create artificial cilia in the magnetic field. The artificial cilium was actuated under the influence of the homogeneous magnetic field. The tilted conical path observed by the artificial cilium was the most uncomplicated asymmetric motion used to pump the fluid. The figure was reproduced with permission from [59], published by the National Academy of Sciences, 2010. (**F**) The performance of the artificial cilia was tested with asymmetric beating trajectories. The figure was reproduced with permission from [109], published by John Wiley and Sons, 2019.

The artificial elastomers were actuated using the pressure sources created by electro-pneumatic actuators. Pneumatically actuated artificial cilia were showcased by Milana and the team [108], where six monolithic PDMS cylinders were offset two times to provide large deflection and swept area. Twelve pressure inputs and pneumatic valves were used to control the six elastomers. Pressure inputs and valves were controlled by the Lab view graphical user interface (GUI). The air inside the elastomer could be diffused with glycerol due to the pressure. The artificial cilia were filled with water to avoid this problem. As a result, the pneumatic signal was converted to hydraulic signals. Figure 2G illustrates the experimental setup of pneumatic actuation. In the same group’s previous study [109], the step-by-step fabrication process of two pneumatic artificial cilia was shown. The two independent pneumatic actuators were used to deliver two degrees of freedom and spatial asymmetry. Figure 3F illustrates the pneumatic actuation technique in which two pneumatic actuators are actuated using dedicated pressure sources. Each actuator has an inflatable cavity body. Similar pneumatic microactuators which were fabricated using the micro-molding processes are reported here [110,111]. Onck et el. [36] established a study where the mixing, pumping, and transport capability of pneumatically actuated artificial cilia were analyzed numerically and experimentally. The study further reported that the antiplectic metachronal wave motion increases the mixing and transportation above and below the ciliary surfaces.

### 4.10. Thermal Actuation Techniques

Recent studies demonstrated that the self-swinging motion of the pendulum could be made by the asymmetrical heat transfer from the pendulum to the immersed medium [112]. Based on this principle, nichrome wire was used as the heat engine to actuate the cilium by Sugioka et al. [113]. The nichrome wire was used as a resistance wire. The artificial cilium was self-swinged in the nucleate boiling regime due to the asymmetrical heat transfer. DC electric charge was sent to the U-shaped wire immersed in the deionized water to set up the nucleate boiling regime. The same group achieved the metachronal wave motion in the low Reynolds number regime [114] using pendulums of different lengths.

### 4.11. Actuation Techniques for Multi-Responsive Artificial Cilia

The ability to respond to multiple stimuli makes artificial cilia known for a wide range of locomotive potential, advanced applications, and functions. The multi-responsive ability leads artificial cilia to next-generation intelligent robots. The cilia body comprises more than one responsive material to response multiple stimuli [115,116]. For instance, Mendes and team [117] fabricated the artificial cilia using electro-responsive gel and magneto responsive nanoparticles. The body of the artificial cilia can deform in the different pH concentrations. As a result of this ability, artificial cilia can be used to detect the pH. Similarly, another artificial cilia array was reported [118], fabricated using the PDMS and CrO_2_ nanoparticles. The study has discussed the multi-responsive technique [119], where apart from actuating artificial cilia through the magnetic field, the shape can be further reconfigured through photo-thermal heating.

## 5. Dynamic Beating Behaviors of Artificial Cilia

The flowing nature created by the beating of artificial cilia can be manipulated by the beating trajectory [51,120]. The dynamic beating behaviors also took an important place in the nonreciprocal nature of cilia, resulting in spatial asymmetry and fluid transportation [7,8]. The beating trajectories were configured and optimized to improve the mixing can be found elsewhere [49,78,92]. The dynamic beating behaviors can be either symmetric or asymmetric by nature. The structures and motion of the motile and primary cilia were illustrated in Figure 3A and Figure 3B, respectively, followed by the symmetric and asymmetric dynamic beating behaviors of artificial cilia.

### 5.1. Symmetric Dynamic Beating Behaviors

Wu et al. [56] demonstrated the symmetric trajectory beating of artificial cilia to achieve both mixing and micropropulsion operations. The observed mixing efficiency and fluid flow rate of micropropulsion operation were 0.84 and 0.089 µL/min, respectively. Chen et al. [50] analyzed the mixing performance for three different trajectories. They are 1. circular, 2. back-and-forth oscillation, and 3. figure-of-eight pattern of artificial cilia. Out of the above three trajectories, figure-eight efficiently provided the mixing performance in the highly viscous fluids. The mixing performance was increased to 0.86 from 0.79 [121]. All the reported beating trajectories were great examples of symmetric dynamic beating behaviors. The symmetric dynamic beating trajectory of the artificial cilium is illustrated in Figure 3C. Figure 3D illustrates two different symmetric trajectories which have been tested to analyze the mixing operation.

### 5.2. Asymmetric Dynamic Beating Behaviors

In contrast to symmetric motion, the studies [59,122] showed that asymmetric motion and improper coordination lead to complex fluid flow. The study of the asymmetric dynamic beating behaviors proved that conical activity is the best and simplest asymmetric nonreciprocal motion [123]. Vilfan et al. [59] demonstrated a nonreciprocal beating by inducing the conical rotation using magnetically actuated artificial cilia. The artificial cilia rotate at the angular frequency of Ꞷ = φ/t. By changing the semi-cone angle, the pumping efficiency of the cilia could be adjusted. Figure 3E illustrates the tilted conical path, which is the best example of the asymmetric motion of the self-assembled cilia.

In low-Re number flow regimes, the fluid propulsion was achieved by the motion asymmetric beating of cilia. Out of a wide range of motion asymmetry, the spatial asymmetry was induced by the difference in the effective and recovery strokes trajectory is challenging to achieve and difficult to control. Milana et al. [109] attained this type of asymmetry using two pneumatic actuators with individual control. The performance of the artificial cilia was tested with asymmetric beating trajectories, as shown in Figure 3F Flow measurements have shown that the two degrees of freedom could generate fluid propulsion in the low Re number regime. It was found that the mixing using the asymmetric motion of artificial cilia was 1.34 times higher than the mixing obtained using the symmetric movement of the artificial cilia [98]. The recent discussions on asymmetric actuation trajectory induced by the artificial cilia can be found elsewhere [58,98].

## 6. Artificial Cilia for Microfluidic Applications

The significant property of microfluidics is that they are capable of delivering practical applications such as mixing, separating, trapping, transporting, and pumping, which involves a small amount of fluid. Passive microfluidic devices such as capillary flow devices might be exploited to get the aforementioned processes done. However, the size and efficiency of such devices are debatable. Integrating an artificial cilia-like setup within microfluidic environment leads to further miniaturization of microfluidic devices. The flexibility in their structural rigidity, actuation capability, and flow generation capability makes artificial cilia as necessary components for the microfluidic devices. Applications of artificial cilia range from lab-on–chip, sensing, particle manipulation, soft robotics, and biomedical devices etc. Several prominent applications in the aforementioned areas are discussed underneath.

### 6.1. Flow Propulsion

Microfluidic channels/devices have difficulties with biochemical reactions due to contamination and limited flow rates. The artificial cilia were positioned within the microchannels to generate regulated fluid flow by actuating them with respect to the external stimuli. For instance, in the study [99], varying the magnetic particle distribution during the artificial cilia fabrication process led to four kinds of versatile flows: circulatory fluid flows, direction-reversible flows, oscillating flows, and pulsatile flows. Besides solving the problems of limited flow rate and contamination, the proposed artificial cilia pump increased the options of fluids to manipulate. In intelligent robots and microfluidic devices, integrating both functional device capable of both sensing and pumping was challenging. Kong et al. [125] demonstrated the self-adaptive magnetic photonic nano-chain cilia arrays to address this issue. The importance of the metachronal wave motion and the asymmetric dynamic beating behavior were briefly explained earlier. The artificial cilia were indulged in the different experimental setups to achieve those characteristics for better pumping performance. Surprisingly, the strategy is different from that in natural cilia. Toonder et al. [62,126] demonstrated two different ways to achieve metachronal wave motion, and their unique benefits were also discussed. First [126], a custom-made magnetic actuation setup provided a non-uniform magnetic field. The metachronal wave was achieved by causing adjacent cilia to move out of phase in the non-uniform magnetic field. The combined effect of inertia force, metachronal and asymmetric wave motion of artificial cilia created the velocity of 3000 µm/s in water medium and 60 µm/s in pure glycerol in the low Reynolds number of 0.05. In the second approach [62], the magnetic artificial cilia were actuated in the uniform magnetic field. However, during the curing process, the magnetic artificial cilia (MAC) array was kept on the group of rod-shaped magnets with an alternating dipole orientation with each neighboring magnets for the remanent magnetization and paramagnetic particle distribution. The metachronal motion was achieved using this strategy during magnetic actuation in the uniform magnetic fields. Apart from pumping capability, the reported MAC, could climb in the slopes from 0° to 180° and carry weighs of 10 times higher than the MAC array. The proposed artificial cilia shed light on the applications of swimming microrobots, biofouling, and on-chip micropumps. In addition, recently, an explicit study [127] established a scientific investigation on the metachronal coordination of the artificial cilia towards the generated fluid flow. In the study, M.Sitti and his team concluded that the antiplectic metachronal coordination waves of the artificial cilia improved the fluid flow. The magnetically actuated artificial cilia of same size as the natural cilia (~10 µm) were fabricated using the microfabrication approach for this study. Concerning the viscosity of the fluid, magnetic torque, and elastic forces of the infinitesimal segment of the artificial cilium, the scaling analysis on the cilia length of the single cilium were conducted. The established study not only proved that the induced flow was enhanced by the integration of the non-reciprocal motion and metachronal coordination but also unveiled the implications of boundary surfaces of artificial cilia, locations of the defects, properties of fluid environment towards the resulted fluid flow in the Re number as same as the biological counterparts of the artificial cilia.

The presence of the artificial cilia in the electroosmotic pumps needs to be noticed very well for future bioinspired thermal micro/nanofluidic technologies. Recently, Saleem et al. [128] numerically analyzed the transportation of thermally radiated nanofluid in the microchannel in which the inside layer was settled with artificial cilia. In similar physical conditions, parameters such as electric potential, directional flow, velocity, pressure, temperature, and entropy generation were numerically analyzed by the same group [129,130,131]. Outside osmotic pumps, a numerical study [132] was reported to examine the heat transfer in the rectangular channel under the influence of the mechanical stirrer or artificial cilia. The study was proposed to find the heat increment or reduction over the increasing Re and Peclet number.

### 6.2. Mixing

**Figure 4:**
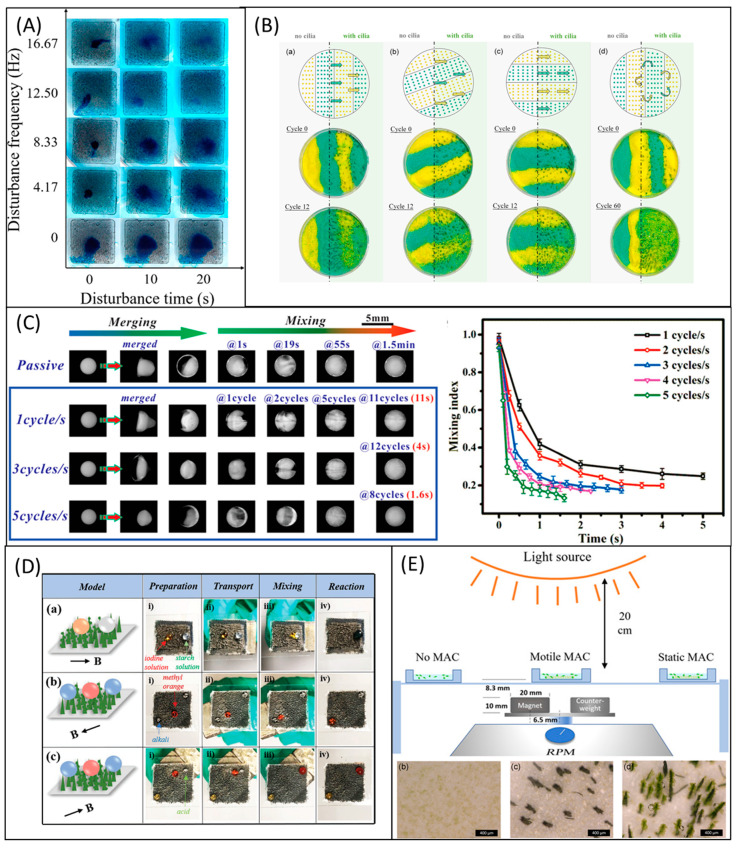
Artificial cilia for mixing. (**A**) Artificial cilia were actuated by magnetic fields in different frequencies to diffuse the blue ink in the water medium. The figure was reproduced with permission from [134], published by the American Chemical Society, 2020. (**B**) The mixing experiment was conducted by the artificial cilia in metachronal motion and nodal-like synchronous motion. Significant mixing performance was achieved by nodal-like synchronous motion barely affected by the arrangement. The figure was reproduced with permission from [135], published by John Wiley and Sons, 2021. (**C**) The mixing performance of the magnetic responsive film shielded by the microlevel artificial cilia was reported here. Complete mixing of fluorescent droplets was achieved in 1.6 s. The relationship between mixing performance and the frequency was plotted (right). The figure was reproduced with permission from [136], published by the American Chemical Society, 2021. (**D**) Under the influence of the magnetic field, microchemical reactions such as transportation and mixing of starch and iodine droplets were demonstrated by artificial cilia. The figure was reproduced with permission from [30], published by John Wiley and Sons, 2021. (**E**) The experimental setup used for microalgae culture embedded with artificial cilia mixing subsets: microalgae growth under (**b**) no cilia (**c**) static magnetic artificial cilia (**d**) motile magnetic artificial cilia. The figure was reproduced with permission from [137], under a Creative Commons BY Non-Commercial No Derivative Works (CC BY-NC-ND 4.0) license, published by John Wiley and Sons, 2021.

The nature of the microfluidic regime is highly different from the microfluidic regime, due to the viscous force dominates the inertia force. Hence, mixing any reagent with the fluid medium is difficult in microfluidic devices. The challenge is to create a perfect/complete mixing in this microfluidic regime. Artificial cilia are well-known actuators for mixing and considered as an active microfluidic device. They have outperformed diffusion and vibration-induced mixers. Studies further reveal that the artificial cilia-based mixers enable the mixing operation more than the passive micromixers [133]. Researchers investigated artificial cilia to improve the mixing performance; as a result, fields from hydrogen production to microalgae growth can benefit from it. In terms of hydrogen production, a study stated the importance of artificial cilia mixing in photocatalytic activity [134]. Photocatalytic activity of g-C_3_N_4_ involves a recycling problem and the efficiency of hydrogen production is low. Researchers incorporated artificial cilia in the photocatalytic process in which graphene oxide (GO) was used as a bridge between the ciliary array and g-C_3_N_4_. Figure 4A illustrates the relationship between the increasing mixing performance and the actuation frequency of artificial cilia over time. Surprisingly, hydrogen production was improved under the influence of actuated artificial cilia by 75% compared with the control group with a static state. Moreover, the problem of recycling was resolved by employing artificial cilia. More on the photocatalytic activity improvement using optimized artificial cilia is discussed later in the photocatalysis section.

Artificial cilia with multifunctional abilities were made to develop bioinspired systems through which a broad spectrum of applications were made [135]. Figure 4B illustrates the result of the mixing experiment conducted by the artificial cilia in metachronal and nodal-like synchronous motions. Significant mixing performance was achieved by nodal-like synchronous motion and the overall performance barely affected by the arrangement.

In biochemical analyses, droplet-based digital microfluidics and medical diagnosis, fluid droplet manipulations such as directional transportation and mixing are necessary. However high-speed mixing, and high-volume droplet transportation are challenging. Zhou et al. [136] fabricated artificial cilia with super-hydrophobicity which were actuated by permanent magnets. Such artificial cilia were demonstrated to achieve complete mixing in ~1.5 s, and it is 60 times improvement compared with typical diffusional mixing. Figure 4C illustrates the mixing performance of the magnetic responsive film shielded by the micro-level artificial cilia and the relationship between mixing performance and frequency. Thanks to the permanent magnet that generated superhydrophobicity without any additional surface salinization, the superhydrophobicity lasted even after all the tests such as pressing, mechanical abrasion, chemical reactions, and sand abrasion. In the concept of acquainting both superhydrophobicity and wettability in the artificial cilia, a novel fabrication approach [30] was introduced by researchers. Figure 4D illustrates chemical reactions such as transportation and mixing of starch and iodine droplets by artificial cilia.

Microalgae such as Scenedesmus subspicatus is an essential product in food, biofuel, and many bio-products. In recent days, microalgae have been cultured in microfluidic channels. A study [137] reported use of magnetic artificial cilia to improve the growth rate of microalgae by creating flow and mixing. Using plasma treatment, hydrophilic artificial cilia were made. The mixing performance induced by the hydrophilic and hydrophobic artificial cilia improved the microalgae growth by ten times and two times, respectively, compared with the control group. The experimental setup used for microalgae culture embedded with artificial cilia mixing was illustrated in Figure 4E. In the figure, the subsets showcased the microalgae growth under (b) no cilia, (c) static artificial cilia (d) motile artificial cilia.

To improve mixing, magnetic artificial cilia were facilitated in the microchannel by researchers, and numerous physical attributes were analyzed both theoretically and experimentally [50,98,138,139]. However, mixing various reagents in the microsized platforms requires real-time adaption in the beating trajectory of artificial cilia. As a result of the issue, researchers intended to control the artificial cilia using fingertip drawing through remote control [138]. The artificial cilia setup was tested with four different trajectories. The built-in method was a perfect figure of eight pattern. The observed results showed the mixing performance of approximately 0.8 in 8 s, which is practically advantageous for a highly viscous medium. In general, Newtonian fluids [140], water and ink [86], silicone oils [78], high viscous dyed solutions [50], DI water and a fluorescent dye [141], colored dye solutions [133], and aqueous glycerol solution [49] were used to calculate the mixing performance by researchers.

### 6.3. Sensing

**Figure 5:**
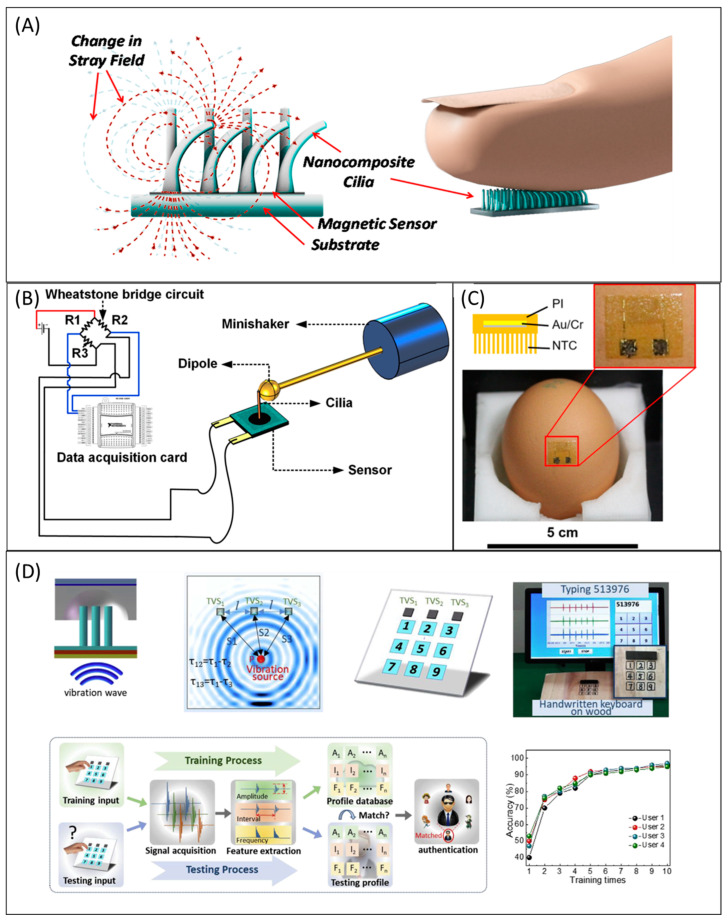
Artificial cilia as sensors. (**A**) Tactile Sensor for harsh environmental conditions. The stray magnetic field of the giant magnetoresistive (GMR) sensor was changed when the artificial cilia were deflected due to touching. The changes in the magnetic field led to the variation in the resistance by which external force was sensed. The figure was reproduced with permission from [152] under a Creative Commons BY (CC-BY) license, published by MDPI, 2016. (**B**) The cilia-inspired flow sensor was comprised of the artificial cilium and the mini shaker. The dielectric was connected to the mini shaker, and it was kept at the ciliary tip in the rest position to find the tip displacement response towards the flow. Triplicate measurements of the tip displacement were conducted when the dielectric was actuated at the constant frequency of 35 Hz. The figure was reproduced with permission from [45], under a Creative Commons BY (CC BY) license, published by MDPI, 2020. (**C**) Photographic image of temperature sensor created and assembled using nanotubular cilia (NTC). PI/Au/Cr/PI with NTC assembled temperature sensor used to find the temperature on the egg (PI: Polyamide, Au: gold, and Cr: Chromium) (**top left**). For comparison, a conventional sensor tested the egg’s temperature when heated using the oven (**bottom**). Images reproduced with permission from [160], published by the American Chemical Society, 2020. (**D**) The schematic illustration of the triboelectric vibration sensor (**top left**). The artificial cilia sensors were demonstrated to be a keyboard (**top left**). The black diagram of the training and testing process for authentication was shown (**bottom left**). The graph plotted training times and the accuracy percentage (**bottom left**). Images reproduced with permission from [161], published by Elsevier, 2019.

Several types of research have been conducted towards the design development of artificial cilia sensors [48,142,143,144,145,146,147,148,149,150,151,152]. The artificial cilia sensors were designed to be used in the marine system’s sensors [153,154], flow sensors [48,155,156], force sensors [150], and tactile and texture sensors. The hydrophone is the ideal example of artificial cilia-based sensors designed and fabricated to use in marine system sensors. Annulus-shaped ciliary hydrophone [153] was recently established by mimicking the neuromasts in lateral fish lines by the researchers. The reported ciliary hydrophone can be used to determine the ship’s motion. The proposed annulus ciliary-shaped sensor outperformed the previously reported bionic cilium-shaped sensor by the same group [154]. In response to the need for flow sensors, Alfadhel et al. 2014 [48] designed and developed a magnetic nanocomposite artificial cilium-like structure on a magnetoimpedance (GMI) thin-film sensor. The ciliary pillars have high elasticity and deflect corresponding to external flow resulting in a net change in mean magnetic intensity value perturbing the impedance of the GMI sensor. By relating the change in impedance to the flow property, the relation was established to quantify the flow. This sensor can detect air and water flow with the sensitivity of 24 mΩ (mm)^−1^ s and 0.9 mΩ (mm)^−1^ s, respectively [48]. The ciliary nanowires can be used as the tactile sensor to sense the touch and flow manipulations which were connected to the giant magneto-impedance (GMI) sensor. When the artificial cilia were touched by flow or humans, the magnetic intensity on the GMI sensor was disturbed, and thus the force could be detected [142,157]. Ribeiro et al. utilized a giant magnetoresistance sensor (GMR) and devised a simulation model to fabricate ciliary force sensors for various robotic applications [146]. Figure 5A illustrates the tactile Sensor for harsh environmental conditions. The stray magnetic field of the giant magnetoresistive (GMR) sensor was changed when the cilia were deflected due to touching. The changes in the magnetic field led to the variation in the resistance by which external force was sensed [152]. In robotics, the skins of robots were expected to have sensitivity with high accuracy; as a result, the robots can have comparable quality to the human skins. A study was proposed where the electronic cilia (EC) [158] could sense both magnetic field and pressure. The reported electronic cilia (EC) were designed and demonstrated to use in e-skin. Researchers claimed that the benefit of the integrated pressure-magnetic field sensor EC is more remarkable than piezoresistive sensors [45,159] and magnetic sensors. Figure 5B illustrates that the cilia-inspired flow sensor comprised the artificial cilium and the mini shaker. Dielectric material was connected to the mini shaker, and it was kept at the ciliary tip in the rest position with the tip displacement response pointing towards the flow. Triplicate measurements of the tip displacement were conducted when the dielectric was actuated at the constant frequency of 35 Hz [45]. Nanotubular cilia with polyimide substrate on the nonpatterned Anode Aluminum Oxide (AAO) templates and Cr and Au deposition by sputtering created a non-harmful flexible temperature sensor which was successfully tested on the eggshell without electrical failure (Figure 5C) [160]. Another study [44] was recently established where the magnetized nanocomposite artificial cilia were embedded with working magnetoresistive sensors. The fabricated sensors sensed the mature level of the strawberries and blueberries nondestructively. Small-sized nano ciliary arrays are sensitive and accurate to transport the vibration with the triboelectric effect. Due to this feature, nanometer ciliary arrays were demonstrated to be used as vibration sensors which were illustrated in Figure 5D as ubiquitous surfaces, human-machine interfaces, and buttonless keyboards such as touching and tapping keyboards for computers and other electronic devices [161]. The sensing principles were summarized along with fabrication and actuation methods in Table S1: Classifications of technologies and actuation/sensing mechanisms in Supplementary Materials.

### 6.4. Contemporary Emerging Applications

#### 6.4.1. Zebrafish Research

**Figure 6:**
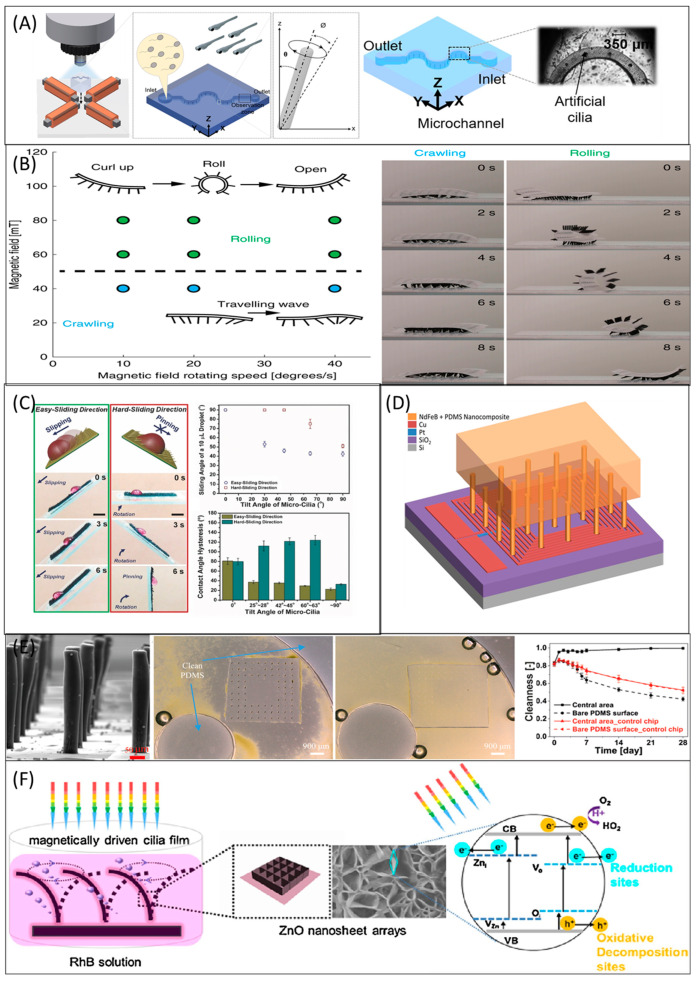
Contemporary emerging applications. (**A**) Illustration of artificial cilia used for Zebrafish research. The magnetic actuation setup was used to control the cilia (**left**). The microfluidic channel embedded the artificial cilia to activate the zebrafish sperms (**second left**). SEM picture of the observation area (**right**). The figure was reproduced with permission from [94], under a Creative Commons BY (CC BY 4.0) license, published by Springer Nature, 2018. (**B**) Artificial cilia embedded minimal robots/Microrobots and soft robots. The photographic images show the crawling and rolling movement of the soft robot (**right**). The graph plotted the magnetic field rotational speed and the magnetic field (**left**). The figure was reproduced with permission from [65], under a Creative Commons BY (CC BY 4.0) license, published by Springer Nature, 2020. (**C**) Artificial cilia surface with wettability. For the sake of wettable property, the slipping and pinning of the droplet on the ciliary array were showcased (**right**). The figure was reproduced with permission from [170], published by John Wiley and Sons, 2017. (**D**) The energy harvester comprised two main components: (**i**) the microfabricated coils on the plane and (**ii**) high aspect ratio (HAR) artificial cilia. The modulus of elasticity of proof mass and HAR of the artificial cilia were endeavored to have the setup to respond to low frequencies. The figure was reproduced with permission from [174], published by John Wiley and Sons, 2018. (**E**) Artificial cilia for antifouling and self-cleaning. The ciliated area was clean compared with the control group (**second from left**). Non-actuated cilia led to fouling, captured after 28 days using fluorescent microscopy (third one from left). The figure was reproduced with permission from [176], under a Creative Commons BY Non-Commercial No Derivative Works (CC BY-NC-ND) license, published by the American Chemical Society, 2020. (**F**) The schematic has illustrated the strategy of artificial cilia for the photocatalysis process. Inner-motile ZnO nanofilm (001) facets were actuated to increase the mass transfer and mixing performance and improve the photocatalytic process. The figure was reproduced with permission from [185], published by Elsevier, 2017.

Zebrafish have a similar genetic structure to humans, and researchers are interested in taking up zebrafish research to the next level by employing artificial cilia for zebrafish research. For instance, the artificial cilia were embedded in the microchannel which was facilitated with a moving wall feature using shape memory alloy to rotate and control the zebrafish stepwise for the benefit of hemodynamic screening [162]. The shape memory alloy-based miniaturized actuator’s detailed fabrication and the controlling process can be found elsewhere [163]. Other recent findings on artificial cilia-assisted zebrafish research can be found elsewhere [52,94,95]. For instance, the artificial cilia in the microfluidic platforms used to activate sperm are shown in Figure 6A [94].

#### 6.4.2. Minimal Robots/Microrobots and Soft Robots

Artificial ciliary arrays have been utilized as soft robots and encoded with both symplectic and antiplectic metachronal wave like motion. Recent research [65] by Nelson et al. 2020 showed (Figure 6B) that the ciliary arrays can be used for particle manipulation in the fluid medium and soft robots in the air medium. The soft robots from the same study can crawl and roll, which can be controlled accurately by the magnetic field and inspired by the giant African millipede. Other recent discussions over artificial cilia embedded robotic engineering can be found elsewhere [164,165,166]. A computational model on Belousov−Zhabotinsky (BZ) cilia to create soft robots oscillating using light for the fluid environments was reported by Balazs et al. [167]. A multi-legged soft millirobot was recently reported by Lu et al. [165]. The magnetic field-guided assembly approach, a template-free fabrication technique, was used to fabricate the robot. In this approach, the magnetic particle was mixed with PDMS and hexane. Under the magnetic field, the tapered feet structures of artificial cilia were produced on the polystyrene substrate. Further, the substrate was kept at 80 °C for 1 h before removing the ciliary robot from the polystyrene substrate to fabricate the robot.

#### 6.4.3. Wearable Devices/Electro-Devices

The modern wearable electro-devices are not heavy but deformable. It requires non-toxic solvents and glue to make “sticky and play” kits. As a result of this specific requirement, the study demonstrated membrane-type flexible and nanotubular cilia to increase the spatial interfacial adhesion on complex shapes such as stone, bark, and textiles [160]. Recently, wearable artificial cilia [168] were fabricated using polymer materials in the form of a microneedle array. The fabricated microneedle array was demonstrated in the mice for psoriasis to be a drug delivery system for transdermal treatments.

#### 6.4.4. Artificial Cilia with Wettability and Hydrophobicity

Hydrophiles/wettability and hydrophobicity are the two physical properties that are endeavored in artificial cilia surfaces. By acquiring these two properties, artificial cilia can manipulate droplets and solid particles in all kinds of mediums and facilitate droplets’ pinning and no pinning options. For example, the study [169] has shown that oil droplets could be transported due to superoleophobicity. The properties can be achieved by various methods such as lubricating, characterizations, and topography modifications by physical and chemical methods. Both permanent and switchable options were encoded [39,170,171,172,173]. The unidirectional wetting properties of the artificial cilia are shown in Figure 6C.

#### 6.4.5. Energy Harvesting

Electromagnetic induction coils were used to harvest the electrical energy transformed from ambient vibrations. The electromagnetic induction coil can have either a stationary coil and moving magnetic flux or a stationary magnetic flux and moving coil. A recent study proposed an artificial cilia embedded unique structure to harvest the energy (Figure 6D), and known as the magnetic composite energy harvester [174]. The energy harvester was comprised of two main components: (i) the microfabricated coils on the plane and (ii) high aspect ratio (HAR) artificial cilia. The modulus of elasticity of proof mass and HAR of the artificial cilia were endeavored to have the setup to respond low frequencies. In a recent study [175], magnetic actuated artificial cilia were prototyped to showcase the waste energy conversion process. The artificial cilia were fabricated using ZnO, which is a piezoelectric material. The artificial cilia harvested the mechanical energy from the flow. The piezoelectric catalysis reaction was triggered by the rotating artificial cilia. As a result of the piezoelectric catalytic performance, Ag nanoparticles were dispersed on the ZnO cilia. The catalytic performance was improved by this process. The mechanical energy was converted into chemical energy, and the study paved the path towards other energy conversions such as hydrogen energy conversion from waste energy.

#### 6.4.6. Antifouling or Self-Cleaning

Fouling is the accumulation of unwanted microparticles in the liquid surfaces, water quality analyzers, marine sensors, and lab-on-a-chip applications. Actuation of the artificial in the microfluidic chip removed 99% of algae, and the study [176] (Figure 6E) shows artificial cilia can be used for antifouling. The level of cleanliness can be found using the simple formula as follows,

(1) $$Cleanliness=1-\left(\frac{A_{Heavy}+\frac{1}{2}A_{Normal}}{A_{Total}}\right)$$

In the total observation area (*A_total_*), *A_heavy_* refers to a heavily affected zone, *A_normal_* refers to a commonly affected zone [176]. In the existing literature on antifouling or self-cleaning, the magnetic artificial cilia employed were several hundred micrometers in length mostly. The dimension limits the application of magnetic artificial cilia to a large scale in microfluidic chips. Recently, an investigation [177] was reported in which the magnetic artificial cilia were sized the same as their biological counterpart which could provide a high degree of cleanliness from 69% in the worst scenarios to almost 100% in the frequency of 40Hz. The other recent discussions over self-cleaning by artificial cilia can be found elsewhere [169,178,179].

#### 6.4.7. Photocatalysis

Photocatalysis attempts a promising approach to create a sustainable society. Some examples are photocatalytic electrolysis of water, solar power generation, carbon capture technology, and pollutant degradation. So, researchers decided to enhance the photocatalytic activity using motile ciliary films. They found that the observed performance is better than the conventional stationery photocatalyst films. Many studies have exposed that artificial cilia with various sizes and dynamic beating behaviors improved photocatalytic activity [134,180]. They address issues such as environmental contamination and energy supply problems. For example, a study [181] shows that the synergic effects of the magnetic artificial cilium, ZnO nanowires, and CdS quantum dots enhanced the H_2_ generation considerably. Several materials, such as TiO_2_, ZnO, CdS, MoS_2_, and BiVO_4,_ have been reported as efficient photocatalyists through testing them in artificial cilia embedded device. It is well known that powder photocatalyst inherently benefits from interior mass transfer in quick succession. As discussed earlier, the artificial cilia-based micromixer can mix rapidly. Hence, the artificial cilia enhanced photocatalytic performance by improving the internal mass transfer [180,182,183,184,185]. The study (Zhang et al.) discussed that researchers combined magnetically actuated artificial cilia and motile photocatalyst film which led to a three-fold improvement in photocatalytic activity compared to traditional planar film [180]. ZnO is a well-known photocatalyst, promised material due to its nontoxicity and thermal stability. Peng et al. established a study where ZnO nanorods were converted to ZnO nanosheets by only increasing the seeding time. The ZnO nanosheets with exposed (001) facets were inserted into the motile inner film by seed-mediated hydrothermal growth strategy for the first time. In the study, (001) facets exposed ZnO nanosheet arrays on the magnetic artificial cilia, and ZnO nanorod arrays film were tested for photocatalytic efficiency. It was realized that ZnO nanosheet arrays with active (001) facets film-coated cilia have 2.4 fold better photolytic performance compared to ZnO nanorods [185]. The schematic (Figure 6F) has illustrated the strategy of artificial cilia for the photocatalysis process. Another study reported that 2D TiO_2_ nanosheet film was associated with 3D magnetic artificial cilium. Under the influence of the magnetic field rotation of 800 rpm yielded RhB degradation considerably [183]. Peng et al. further designed a photocatalytic film that integrated the BiVO_4_, ZnO, and magnetic artificial cilia to increase mass transfer, light absorption, and photocatalytic activity [182]. Another recent study [93] showed the possibility of expanding the photodegradation capability by changing the arrangement of artificial cilia.

#### 6.4.8. Particle Manipulation

Particle manipulation is the process where single or multiple solid particles or liquid droplets are transported in the sub-microscale fluid medium by artificial cilia. Droplet-based microfluidics involves the manipulations of (a) water droplets and (b) oil droplets. Strong observations of droplet generation, merging, separation, and sorting are required for such droplet manipulation. On the other hand, solid particle-based microfluidics involves the manipulations of (c) polymer or viscoelastic particles.

(a) Water droplets manipulation

In water droplet manipulation, single or multiple water droplets were transported in the liquid or air medium by the ubiquitous established strategies by the artificial cilia. Magnetically responsive artificial cilia were primarily implemented to direct the water droplets in the microfluidic device. Tilting angle and contact angle from the surface are the predominant parameters of the artificial cilia to control the droplets. Those angles by means of bending deflections were adjusted using the magnetic field. In addition, the ciliary body was enhanced with advanced necessity surface properties such as hydrophobic and wettability, optical properties, and intrinsic body materials to control the droplets. The investigation [172] discussed a novel droplet transporting using the ferromagnetic artificial cilia. In the study, the artificial cilia were upgraded with the switchable hydrophobicity wettability, and the surface properties. The wettability and adhesion can be switched reversely by applying the magnetic field. The on/off control of the magnetic field was utilized to bring back the adhesion and surface sliding consecutively to transport the droplets. If the magnetic field was switched off, the surface would become water-repellent and if the magnetic field was switched on the surface would become water-adhesive.

**Figure 7:**
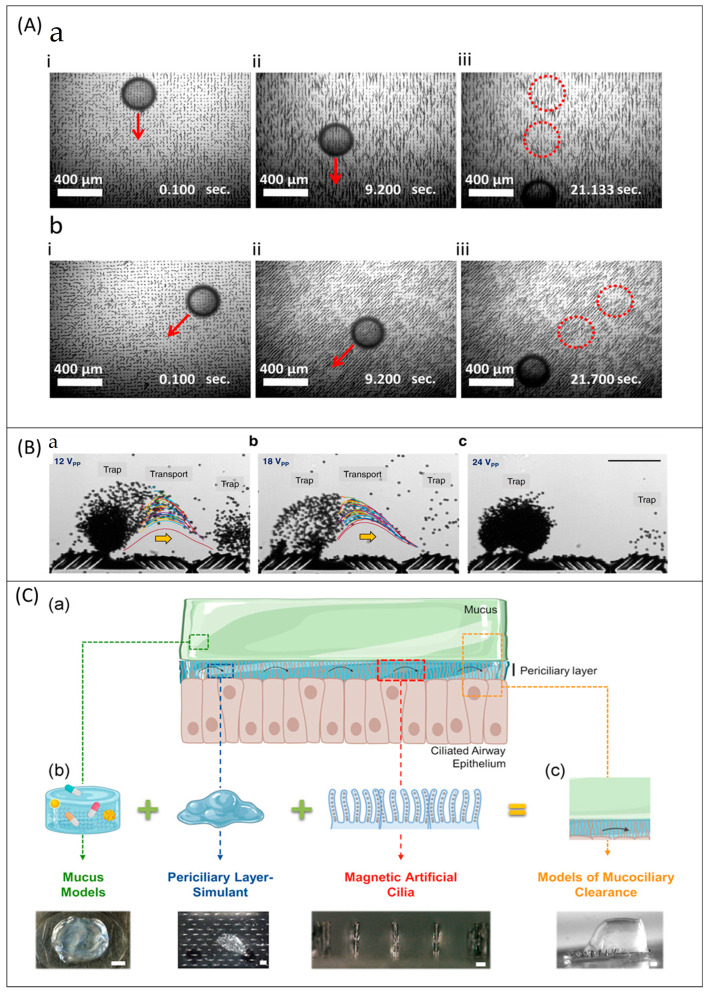
Artificial cilia for particle manipulation. **(A**) The transportation of the water droplet by artificial ciliary lubricated surfaces was shown in the schematics. Filling in the silicone oil led to the lubricated surfaces of the magnetic responsive arrays. The figure was reproduced with permission from [189], under a Creative Commons BY (CC BY) license, published by AIP Publishing, 2020. (**B**) The transport and trapping capability of artificial ciliary bands were showcased. By changing the acoustic-electric field, both transporting and trapping beads in the aqueous medium were achieved by the same microarchitecture of the ciliary band. The figure was reproduced with permission from [29], under a Creative Commons BY (CC BY 4.0) license, published by Springer Nature, 2021. (**C**) Illustration of modeling human mucus transportation. As a milestone in the viscoelastic particle transportation by artificial cilia, physiological and pathological mucus were modeled using L-Mu^3^Gel and CF-Mu^3^Gel and achieved transportation in the respiratory airway model by artificial cilia. The figure was reproduced with permission from [188], published by John Wiley and Sons, 2021.

Instead of depending upon the magnetic field ultimately, hydrophobicity and slippery properties were achieved on the surfaces of artificial cilia by infusing them in the lubricants. For instance, the artificial cilia [186] were fabricated using PDMS and carbonyl iron microspheres. The polystyrene (PS) nanoparticles were deposited on the ciliary body. Thus, it was comprised of a hierarchical structure. The water droplet was pinned by the hydrophobic property of the ciliary tip. The infused lubricant (perfluorinated oils) indulged in the slipperiness of the droplet when the ciliary body was tilted due to the exerted magnetic field. In the same year, a similar slippery approach was used in another study [170]. Figure 7A illustrates the transportation of the water droplet by artificial ciliary lubricated surfaces. The lubricated surface of the magnetic responsive arrays was achieved by filling in the silicone oil. In the uniform magnetic field, the entire ciliary array responded to the magnetic field.

On the other hand, a single row or column of artificial cilia responded to the dual magnets comprised junction induced magnetic field, which can create precise unidirectional wave motion. Double the magnet junctions have been introduced as a spectacular milestone in droplet manipulation [37,39]. Song et al. [39] demonstrated that the artificial cilia that moved the water droplet using dual magnets comprised junctions induced unidirectional wave motion using a single column of the ciliary array. The single droplet was identified to travel in the straight line and arc orbit whereas two droplets were moved in the parallel trajectories and mixed in the single orbit.

(b) Oil droplets manipulation

Oil droplet manipulation has been getting attention recently in material processes, anti-fouling, and self-cleaning. For example, Zhang and his team [171] fabricated artificial cilia with mushroom head microstructures. The artificial cilia were showcased the amphiphilic ability by manipulating oil droplets in the water medium and water droplets in the air medium by switching reverse wettability. Importantly, no lubricant treatment was required for such artificial cilia. Another study [169] demonstrated the oleophobic ability of the artificial microcilia by transporting oil droplets in air and underwater by transforming the surface into hydrophobic.

(c) Polymer or viscoelastic particles manipulation

Over many years, the transporting mechanics of the artificial cilia were improved drastically, yet there is a lack of innovation towards the transportation of the viscous/polymer particles. The conical trajectory movement of the beating cilia, changing shapes, and the frictional forces of the beating artificial cilia are the strategies behind the manipulations of the viscous/polymer particles [38,178]. The relationship between particle transportation and the ratio of particle size to cilia pitch was reported in the study [187]. A recent study [38] ascertained the manipulation of polylactic acid (PLA) particle transportation in air and water using the magnetically actuated artificial cilia. It was concluded that the adhesive and the frictional forces between the artificial cilia and the moving particles are necessary for particle transportation. The transportation was achieved in the direction of the tilted conical movement and the effective stroke direction [38]. Figure 7B showcased the transport and trapping capability of artificial ciliary bands. By changing the acoustic-electric field, both transporting and trapping beads in the aqueous medium were achieved by the same microarchitecture of the ciliary band. Pacheco et al. [188] proposed a study where the modular materials such as L-Mu^3^Gel and CF-Mu^3^Gel, equivalent to the physiological and CF mucus, respectively, were transported successfully with the magnetic artificial cilia. The artificial mucus models L-Mu^3^Gel and CF-Mu^3^Gel can be created using readily available materials. The study helps to understand the drug processing time in the mucus clearance ciliary region. Figure 7C illustrates the modeling of human mucus transportation.

## 7. Conclusions and Future Directions

Artificial cilia have been developed to be implicit dominant tools for microfluidic applications. Certain research groups have sought to increase artificial cilia’s efficiency, especially in providing micromixing, pumping, and particle handling applications for microfluidic purposes. In addition, artificial cilia have been enriched with the ability to carry, sense, communicate and locomote. This review briefly discussed the concepts from the current developments, fabrication processes, actuation strategies, dynamic beating behaviors, functionalities, uniqueness, the potential applications, deliverables, remaining challenges, and the future trends by biomimetic cilia. The advantages and disadvantages of each strategy have been discussed explicitly.

Bringing artificial cilia to commercial applications has been challenging since the work was started. Upgrading the artificial cilia abilities has been exceptionally vital for understanding the mechanisms that can be utilized in the future. Cost-effective and efficient manufacturing strategies must be set up to deliver artificial cilia to more actual utilization in lab-on-a-chip gadgets. Even though the natural cilia are highly different from today’s biomimetic cilia, advances in technology will undoubtedly produce artificial cilia that are close to the natural cilia and generate a large variety of new and exciting achievements in the future.

## Supplementary Materials

The following are available online at https://www.mdpi.com/article/10.3390/mi13050735/s1, Table S1: Classifications of technologies and actuation/sensing mechanisms.
