# Supplementary material for: Microfluidic Applications of Artificial Cilia: Recent Progress, Demonstration, and Future Perspectives
Source: Micromachines (Basel). 2022 May 3;13(5):735. doi: 10.3390/mi13050735 (PMC9147031; doi:10.3390/mi13050735)
Supplement: Supplementary file 1 [file micromachines-13-00735-s001.zip › micromachines-1672284-supplementary.pdf]

Supplementary Materials

Microfluidic Applications of Artificial Cilia: Recent Progress, Demonstration, and Future Perspectives

Vignesh Sahadevan[1, †], Bivas Panigrahi[2, †], and Chia-Yuan Chen[1,*]

Table S1. Classifications of technologies and actuation/sensing mechanisms.

Fabrication methods	Actuation methods	Sensing mechanisms
Micro-molding fabrication techniques [1–8]	Optical actuation techniques [9,10]	Piezoresistive principle [11–21]
Photolithography fabrication techniques [22–24]	Electrostatic actuation techniques [25,26]	Magnetoresistive principle [22,27–31]
3D/4D/5D printing fabrication techniques [11,12,14,32,33]	pH actuation techniques [34-36]	Magnetoimpedance principle [37–39]
Facile bottom-up approaches [40,41]	Resonance actuation techniques [42]	Mechanocapacitive principle [43]
Roll-pulling approaches [44]	Pneumatical actuation techniques [45–47]	Triboelectric based [48]
Self-assembly fabrication techniques [49]	Electromagnetic actuation [8,50,51]	Electronic based [52]
Field-Effect Spinning approaches [53]	Permanent magnetic actuation [54]	
Dip-coating fabrication techniques [55]	Acoustic actuation techniques [56]	
	Electric stimulation actuation techniques [55,57]	
	Induced charge electro-osmosis using AC electric field techniques [58–61]	
	Thermal actuation techniques [62,63]	
	Actuation techniques for multi-responsive artificial cilia [64-66]	

References

1. Chen, C.-Y.; Hsu, C.-C.; Mani, K.; Panigrahi, B. Hydrodynamic influences of artificial cilia beating behaviors on micromixing. *Chemical Engineering and Processing: Process Intensification* **2016**, *99*, 33-40, doi:10.1016/j.cep.2015.10.023.

2. Chen, C.Y.; Chen, C.Y.; Lin, C.Y.; Hu, Y.T. Magnetically actuated artificial cilia for optimum mixing performance in microfluidics. *Lab Chip* **2013**, *13*, 2834-2839, doi:10.1039/c3lc50407g.

3. Chen, C.Y.; Cheng, L.Y.; Hsu, C.C.; Mani, K. Microscale flow propulsion through bioinspired and magnetically actuated artificial cilia. *Biomicrofluidics* **2015**, *9*, 034105, doi:10.1063/1.4921427.

4. Huang, P.-Y.; Panigrahi, B.; Lu, C.-H.; Huang, P.-F.; Chen, C.-Y. An artificial cilia-based micromixer towards the activation of

zebrafish sperms. *Sensors and Actuators B: Chemical* **2017**, *244*, 541-548, doi:10.1016/j.snb.2016.12.113.

5. Lu, C.-H.; Tang, C.-H.; Ghayal, N.; Panigrahi, B.; Chen, C.-Y.; Chen, C.-Y. On the improvement of visible-responsive photodegradation through artificial cilia. *Sensors and Actuators A: Physical* **2019**, *285*, 234-240, doi:10.1016/j.sna.2018.10.045.
6. Wu, Y.-A.; Panigrahi, B.; Chen, C.-Y. Hydrodynamically efficient micropropulsion through a new artificial cilia beating concept. *Microsystem Technologies* **2017**, *23*, 5893-5902, doi:10.1007/s00542-017-3428-3.
7. Panigrahi, B.; Sahadevan, V.; Chen, C.-Y. Shape-programmable artificial cilia for microfluidics. *iScience* **2021**, *24*, 103367, doi:https://doi.org/10.1016/j.isci.2021.103367.
8. Wu, Y.A.; Panigrahi, B.; Lu, Y.H.; Chen, C.Y. An Integrated Artificial Cilia Based Microfluidic Device for Micropumping and Micromixing Applications. *Micromachines (Basel)* **2017**, *8*, doi:10.3390/mi8090260.
9. Van Oosten, C.L.; Bastiaansen, C.W.; Broer, D.J. Printed artificial cilia from liquid-crystal network actuators modularly driven by light. *Nature materials* **2009**, *8*, 677-682.
10. Gelebart, A.H.; Mc Bride, M.; Schenning, A.; Bowman, C.N.; Broer, D.J. Photoresponsive Fiber Array: Toward Mimicking the Collective Motion of Cilia for Transport Applications. *Advanced Functional Materials* **2016**, *26*, 5322-5327, doi:10.1002/adfm.201601221.
11. Kamat, A.M.; Pei, Y.; Kottapalli, A.G. Bioinspired cilia sensors with graphene sensing elements fabricated using 3D printing and casting. *Nanomaterials* **2019**, *9*, 954.
12. Kamat, A.M.; Pei, Y.; Jayawardhana, B.; Kottapalli, A.G.P. Biomimetic soft polymer microstructures and piezoresistive graphene mems sensors using sacrificial metal 3D printing. *ACS applied materials & interfaces* **2021**, *13*, 1094-1104.
13. Sengupta, D.; Trap, D.; Kottapalli, A.G.P. Piezoresistive Carbon Nanofiber-Based Cilia-Inspired Flow Sensor. *Nanomaterials* **2020**, *10*, doi:10.3390/nano10020211.
14. Kamat, A.M.; Zheng, X.; Jayawardhana, B.; Kottapalli, A.G.P. Bioinspired PDMS-graphene cantilever flow sensors using 3D printing and replica moulding. *Nanotechnology* **2020**, *32*, 095501.
15. Asadnia, M.; Kottapalli, A.G.P.; Karavitaki, K.D.; Warkiani, M.E.; Miao, J.M.; Corey, D.P.; Triantafyllou, M. From Biological Cilia to Artificial Flow Sensors: Biomimetic Soft Polymer Nanosensors with High Sensing Performance. *Scientific Reports* **2016**, *6*, doi:10.1038/srep32955.
16. Slinker, K.A.; Kondash, C.; Dickinson, B.T.; Baur, J.W. CNT-Based Artificial Hair Sensors for Predictable Boundary Layer Air Flow Sensing. *Advanced Materials Technologies* **2016**, *1*, doi:10.1002/admt.201600176.
17. Zhang, X.; Shen, N.; Xu, Q.; Pei, Y.; Lian, Y.; Wang, W.; Zhang, G.; Zhang, W. Design and implementation of anulus-shaped ciliary structure for four-unit MEMS vector hydrophone. *International Journal of Metrology and Quality Engineering* **2021**, *12*, 4.
18. Zhang, X.Y.; Xu, Q.D.; Zhang, G.J.; Shen, N.X.; Shang, Z.Z.; Pei, Y.; Ding, J.W.; Zhang, L.S.; Wang, R.X.; Zhang, W.D. Design and analysis of a multiple sensor units vector hydrophone. *Aip Advances* **2018**, *8*, doi:10.1063/1.5044673.
19. Fan, Z.F.; Chen, J.; Zou, J.; Bullen, D.; Liu, C.; Delcomyn, F. Design and fabrication of artificial lateral line flow sensors. *Journal of Micromechanics and Microengineering* **2002**, *12*, 655-661, doi:10.1088/0960-1317/12/5/322.
20. Qualtieri, A.; Rizzi, F.; Epifani, G.; Ernits, A.; Kruusmaa, M.; De Vittorio, M. Parylene-coated bioinspired artificial hair cell for liquid flow sensing. *Microelectronic Engineering* **2012**, *98*, 516-519, doi:10.1016/j.mee.2012.07.072.
21. Chen, N.N.; Tucker, C.; Engel, J.M.; Yang, Y.C.; Pandya, S.; Liu, C. Design and characterization of artificial haircell sensor for flow sensing with ultrahigh velocity and angular sensitivity. *Journal of Microelectromechanical Systems* **2007**, *16*, 999-1014, doi:10.1109/jmems.2007.902436.
22. Carvalho, M.; Ribeiro, P.; Romão, V.; Cardoso, S. Smart fingertip sensor for food quality control: fruit maturity assessment with a magnetic device. *Journal of Magnetism and Magnetic Materials* **2021**, 168116.

23. Dillinger, C.; Nama, N.; Ahmed, D. Starfish-inspired Ultrasound Ciliary Bands for Microrobotic Systems. **2021**.
24. Zhang, S.; Cui, Z.; Wang, Y.; den Toonder, J. Metachronal μ-Cilia for On-Chip Integrated Pumps and Climbing Robots. *ACS applied materials & interfaces* **2021**, *13*, 20845-20857.
25. den Toonder, J.; Bos, F.; Broer, D.; Filippini, L.; Gillies, M.; de Goede, J.; Mol, T.; Reijme, M.; Talen, W.; Wilderbeek, H. Artificial cilia for active micro-fluidic mixing. *Lab on a Chip* **2008**, *8*, 533-541.
26. Minami, K.; Yano, S. Electrostatic Micro Actuator with Distributed Ciliary Electrodes (E-Maccel). *IEEJ Transactions on Sensors and Micromachines* **2004**, *124*, 381-386.
27. Alfadhel, A.; Khan, M.A.; de Freitas, S.C.; Kosel, J. Magnetic Tactile Sensor for Braille Reading. *Ieee Sensors Journal* **2016**, *16*, 8700-8705, doi:10.1109/jsen.2016.2558599.
28. Ribeiro, P.; Khan, M.A.; Alfadhel, A.; Kosel, J.; Franco, F.; Cardoso, S.; Bernardino, A.; Schmitz, A.; Santos-Victor, J.; Jamone, L. Bioinspired Ciliary Force Sensor for Robotic Platforms. *Ieee Robotics and Automation Letters* **2017**, *2*, 971-976, doi:10.1109/lra.2017.2656249.
29. Alfadhel, A.; Khan, M.A.; Cardoso, S.; Kosel, J. A single magnetic nanocomposite cilia force sensor. In Proceedings of the 2016 IEEE Sensors Applications Symposium (SAS), 2016; pp. 1-4.
30. Schroeder, P.; Schotter, J.; Shoshi, A.; Eggeling, M.; Bethge, O.; Hutten, A.; Bruckl, H. Artificial cilia of magnetically tagged polymer nanowires for biomimetic mechanosensing. *Bioinspiration & Biomimetics* **2011**, *6*, doi:10.1088/1748-3182/6/4/046007.
31. Alfadhel, A.; Khan, M.A.; Cardoso, S.; Leitao, D.; Kosel, J. A Magnetoresistive Tactile Sensor for Harsh Environment Applications. *Sensors* **2016**, *16*, doi:10.3390/s16050650.
32. Liu, F.; Alici, G.; Zhang, B.; Beirne, S.; Li, W. Fabrication and characterization of a magnetic micro-actuator based on deformable Fe-doped PDMS artificial cilium using 3D printing. *Smart Materials and Structures* **2015**, *24*, 035015.
33. Gu, H.; Boehler, Q.; Cui, H.; Secchi, E.; Savorana, G.; De Marco, C.; Gervasoni, S.; Peyron, Q.; Huang, T.-Y.; Pane, S. Magnetic cilia carpets with programmable metachronal waves. *Nature communications* **2020**, *11*, 1-10.
34. den Toonder, J.M.J.; Onck, P.R. Microfluidic manipulation with artificial/bioinspired cilia. *Trends in Biotechnology* **2013**, *31*, 85-91, doi:10.1016/j.tibtech.2012.11.005.
35. Glazer, P.; Leuven, J.; An, H.; Lemay, S.; Mendes, E. Hydrogel-based multi-stimuli responsive cilia. In Proceedings of the 2013 NSTI Nanotechnology Conference and Expo, Nanotech 2013, 2013; pp. 138-141.
36. Zarzar, L.D.; Kim, P.; Aizenberg, J. Bio-inspired Design of Submerged Hydrogel-Actuated Polymer Microstructures Operating in Response to pH. *Advanced Materials* **2011**, *23*, 1442-1446, doi:10.1002/adma.201004231.
37. Alfadhel, A.; Li, B.; Zaher, A.; Yassine, O.; Kosel, J. A magnetic nanocomposite for biomimetic flow sensing. *Lab on a Chip* **2014**, *14*, 4362-4369.
38. Alfadhel, A.; Kosel, J. Magnetic Nanocomposite Cilia Tactile Sensor. *Advanced Materials* **2015**, *27*, 7888-7892, doi:10.1002/adma.201504015.
39. Alfadhel, A.; Kosel, J. Magnetic micropillar sensors for force sensing. In Proceedings of the 2015 IEEE Sensors Applications Symposium (SAS), 2015; pp. 1-4.
40. Timonen, J.V.; Johans, C.; Kontturi, K.s.; Walther, A.; Ikkala, O.; Ras, R.H. A facile template-free approach to magnetodriven, multifunctional artificial cilia. *ACS applied materials & interfaces* **2010**, *2*, 2226-2230.
41. Sanchez, T.; Welch, D.; Nicastro, D.; Dogic, Z. Cilia-like beating of active microtubule bundles. *Science* **2011**, *333*, 456-459.
42. Oh, K.; Smith, B.; Devasia, S.; Riley, J.J.; Chung, J.H. Characterization of mixing performance for bio-mimetic silicone cilia. *Microfluidics and Nanofluidics* **2010**, *9*, 645-655, doi:10.1007/s10404-010-0578-3.
43. Sarlo, R.; Leo, D. Airflow sensing with arrays of hydrogel supported artificial hair cells. In Proceedings of the Smart Materials,

Adaptive Structures and Intelligent Systems, 2015; p. V002T006A008.

44. Wang, Y.; den Toonder, J.; Cardinaels, R.; Anderson, P. A continuous roll-pulling approach for the fabrication of magnetic artificial cilia with microfluidic pumping capability. *Lab on a Chip* **2016**, *16*, 2277-2286.
45. Milana, E.; Zhang, R.; Vetrano, M.R.; Peerlinck, S.; De Volder, M.; Onck, P.R.; Reynaerts, D.; Gorissen, B. Metachronal patterns in artificial cilia for low Reynolds number fluid propulsion. *Science advances* **2020**, *6*, eabd2508.
46. Milana, E.; Gorissen, B.; Peerlinck, S.; De Volder, M.; Reynaerts, D. Artificial soft cilia with asymmetric beating patterns for biomimetic low-Reynolds-number fluid propulsion. *Advanced Functional Materials* **2019**, *29*, 1900462.
47. Zhang, R.; den Toonder, J.; Onck, P.R. Transport and mixing by metachronal waves in nonreciprocal soft robotic pneumatic artificial cilia at low Reynolds numbers. *Phys. Fluids* **2021**, *33*, 092009.
48. He, Q.; Wu, Y.F.; Feng, Z.P.; Sun, C.C.; Fan, W.J.; Zhou, Z.H.; Meng, K.Y.; Fan, E.D.; Yang, J. Triboelectric vibration sensor for a human-machine interface built on ubiquitous surfaces. *Nano Energy* **2019**, *59*, 689-696, doi:10.1016/j.nanoen.2019.03.005.
49. Wang, Y.; Gao, Y.; Wyss, H.; Anderson, P.; den Toonder, J. Out of the cleanroom, self-assembled magnetic artificial cilia. *Lab on a Chip* **2013**, *13*, 3360-3366.
50. Chen, C.-Y.; Lin, C.-Y.; Hu, Y.-T. Inducing 3D vortical flow patterns with 2D asymmetric actuation of artificial cilia for high-performance active micromixing. *Experiments in Fluids* **2014**, *55*, doi:10.1007/s00348-014-1765-x.
51. Chen, C.-Y.; Chang Chien, T.-C.; Mani, K.; Tsai, H.-Y. Axial orientation control of zebrafish larvae using artificial cilia. *Microfluidics and Nanofluidics* **2016**, *20*, doi:10.1007/s10404-015-1668-z.
52. Liu, Y.F.; Fu, Y.F.; Li, Y.Q.; Huang, P.; Xu, C.H.; Hu, N.; Fu, S.Y. Bio-inspired highly flexible dual-mode electronic cilia. *Journal of Materials Chemistry B* **2018**, *6*, 896-902, doi:10.1039/c7tb03078a.
53. Jeong, W.; Jeong, S.M.; Lim, T.; Hang, C.Y.; Yang, H.; Lee, B.W.; Park, S.Y.; Ju, S. Self-Emitting Artificial Cilia Produced by Field Effect Spinning. *Acs Applied Materials & Interfaces* **2019**, *11*, 35286-35293, doi:10.1021/acsami.9b09571.
54. Hanasoge, S.; Ballard, M.; Hesketh, P.J.; Alexeev, A. Asymmetric motion of magnetically actuated artificial cilia. *Lab on a Chip* **2017**, *17*, 3138-3145.
55. Becker, K.P.; Chen, Y.F.; Wood, R.J. Mechanically Programmable Dip Molding of High Aspect Ratio Soft Actuator Arrays. *Advanced Functional Materials* **2020**, *30*, doi:10.1002/adfm.201908919.
56. Orbay, S.; Ozcelik, A.; Bachman, H.; Huang, T.J. Acoustic actuation of in situ fabricated artificial cilia. *Journal of Micromechanics and Microengineering* **2018**, *28*, 025012.
57. Dai, B.; Li, S.H.; Xu, T.L.; Wang, Y.F.; Zhang, F.L.; Gu, Z.; Wang, S.T. Artificial Asymmetric Cilia Array of Dielectric Elastomer for Cargo Transportation. *Acs Applied Materials & Interfaces* **2018**, *10*, 42979-42984, doi:10.1021/acsami.8b13419.
58. Sugioka, H.; Ishikawa, M. Artificial carbon cilium using induced charge electro-osmosis. *Aip Advances* **2020**, *10*, doi:10.1063/1.5143700.
59. Sugioka, H.; Ishikawa, M.; Kado, T. Selective Carbon Self-wiring from a Graphite Rod in Water under a DC Electric Field. *Journal of the Physical Society of Japan* **2020**, *89*, doi:10.7566/jpsj.89.024801.
60. Sugioka, H.; Nakano, N.; Mizuno, Y. High-Speed Periodic Beating Motion of a Spiral Gold Thread Using Induced Charge Electro-Osmosis with a Two-Electrode Structure. *Journal of the Physical Society of Japan* **2019**, *88*, doi:10.7566/jpsj.88.084801.
61. Sugioka, H.; Yoshijima, H. Metachronal motion of artificial cilia using induced charge electro-osmosis. *Colloids and Surfaces a-Physicochemical and Engineering Aspects* **2021**, *626*, doi:10.1016/j.colsurfa.2021.127023.
62. Sugioka, H.; Tanaka, M. Thermally Actuated Elastic Cilium Based on the Self-Oscillation Phenomenon Due to Spontaneous Asymmetrical Heat Transfer. *Journal of the Physical Society of Japan* **2022**, *91*, 044402, doi:10.7566/JPSJ.91.044402.
63. Sugioka, H.; Kubota, M.; Tanaka, M. High-Speed Asymmetric Motion of Thermally Actuated Cilium. *Journal of the Physical*

Society of Japan **2020**, *89*, 114402.

64. Glazer, P.J.; Leuven, J.; An, H.; Lemay, S.G.; Mendes, E. Multi-Stimuli Responsive Hydrogel Cilia. *Advanced Functional Materials* **2013**, *23*, 2964-2970, doi:10.1002/adfm.201203212.
65. Li, M.; Kim, T.; Guidetti, G.; Wang, Y.; Omenetto, F.G. Optomechanically Actuated Microcilia for Locally Reconfigurable Surfaces. *Advanced Materials* **2020**, *32*, doi:10.1002/adma.202004147.
66. Liu, J.A.C.; Evans, B.A.; Tracy, J.B. Photothermally Reconfigurable Shape Memory Magnetic Cilia. *Advanced Materials Technologies* **2020**, *5*, doi:10.1002/admt.202000147.
